# Enhanced DWT-OFDM communication system using wavelet domain equalizer with Co-CFO

**DOI:** 10.1371/journal.pone.0317097

**Published:** 2025-04-17

**Authors:** Khaled Ramadan, Emad S. Hassan

**Affiliations:** 1 Department of Telecommunication Engineering, Collage of Engineering at Ahlia University, Manama, Bahrain; 2 Department of Communications and Computer Engineering, The Higher Institute of Engineering at Al-Shorouk City, Cairo, Egypt; 3 Department of Electrical and Electronics Engineering, College of Engineering and Computer Science, Jazan University, Jizan, Saudi Arabia; Ozyegin University: Ozyegin Universitesi, TÜRKIYE

## Abstract

For the design of Orthogonal Frequency Division Multiplexing (OFDM), the Discrete Wavelet Transform (DWT) offers significant benefits over the classic Discrete Fourier Transform (DFT). This article proposes a Joint Low Complexity Regularized Zero Forcing-Wavelet Domain Equalizer (JLCRLZF-WDE) as substitute to a Frequency Domain Equalizer (FDE) for improving the Multiple-Input-Multiple-Output (MIMO) DWT-OFDM. The results have been assessed using the Co-Carrier Frequency Offset (Co-CFO) Rayleigh fading channel. The proposed scheme’s relevance is demonstrated by a comparison of its Bit-Error-Rate (BER) and simulated time with those of conventional schemes. The obtained results showed that the proposed JLCRLZF-WDE outperforms conventional equalizers, requiring only 0.82 dB additional SNR to match the BER performance of Linear Minimum Mean Square Error (LMMSE)-WDE at BER =  10^-3^, and 0.27 dB at BER =  10^-4^. In comparison, other equalizers, such as Linear Zero Forcing (LZF)-FDE and LMMSE-FDE based on DWT and DFT, require significantly higher SNR values to reach the same performance benchmarks, with differences ranging from 0.31 dB to over 15.35 dB. Additionally, the proposed scheme achieves a simulated time reduction of 3.17% and 12.4% compared to LMMSE-WDE based on DWT and DFT, respectively.

## I. Introduction

Because of its effective use of spectrum and resilience to multipath interference, orthogonal frequency division multiplexing, or OFDM, is a crucial technology found in many contemporary wireless communication networks, including Wi-Fi, 4G, and 5G. By partitioning the whole bandwidth into many orthogonal subcarriers, each one is assigned to transmit a segment of the data, OFDM considerably lowers Inter-Symbol Interference (ISI) in wireless situations that is brought on by signal reflections. In contrast to conventional Frequency Division Multiplexing (FDM), OFDM guarantees interference-free frequency overlap between subcarriers, enhancing spectral efficiency [1]. The primary advantage of OFDM is its ability to effectively handle frequency-selective fading, which makes it perfect for wireless broadband applications. Since each OFDM subcarrier is modulated individually, the system is more robust to channel impairments when the data is distributed over a large number of subcarriers. Moreover, OFDM employs a Cyclic Prefix (CP), a duplicate of the signal’s last segment to lessen the impact of multipath delay spread and provide resilience against ISI [1]. OFDM is very helpful in the context of 4G and 5G networks as it allows for huge interconnection and high data rates [2]. Additionally, its ability to handle multipath fading and provide efficient spectrum utilization makes OFDM an ideal candidate for enhancing the performance of Wireless Sensor Networks (WSNs), particularly in scenarios requiring reliable and scalable communication [3]. Moreover, OFDM performs much better when combined with MIMO technology, which multiplexes data spatially. Another advantage is that, in comparison to time-domain equalization techniques, it has a very straightforward equalization procedure in the FD, greatly reducing receiver complexity.

Nevertheless, there are several drawbacks to OFDM, such as a large Peak-to-Average Power Ratio (PAPR) that may result in power amplifier performance that is not very efficient. Many methods are employed to lower PAPR, including filtering and clipping, although these can cause signal distortion. Despite these difficulties, OFDM is a popular technique in wireless networks because its benefits in multipath situations exceed its disadvantages [2]. To further highlight OFDM’s adaptability, it is the foundation for several contemporary broadcasting technologies, such as Digital Audio Broadcasting (DAB) and Digital Video Broadcasting (DVB). The fact that OFDM can handle reflections and signal interference to ensure dependable data transmission is one of the reasons it works so well in crowded, metropolitan settings. Furthermore, OFDM can optimize throughput by adjusting data rates in response to channel circumstances thanks to its adaptive modulation and coding techniques.

Applications for 5G and other wireless communication technologies can also make advantage of this technology. Additionally, the Inverse Discrete Fourier Transform (IDFT) and DFT can used to build a standard OFDM system [1]. The OFDM system may be built utilizing an orthogonal transform such as Inverse DWT (IDWT) at the transmitter and DWT at the receiver [4]. Wavelet-based MIMO-OFDM is increasingly recognized for its potential in wireless communication due to its ability to provide high spectral efficiency, cost-effectiveness, and reduced phase noise [5]. Studies highlight that the DWT enhances system performance compared to the DFT by reducing PAPR, improving non-linearity [6], and overcoming limitations of traditional Multi-Carrier Modulation (MCM) systems [7]. Research demonstrates the effectiveness of DWT in applications such as MIMO-OFDM for wireless communication [8], improving system performance [9], millimeter-wave systems [10], and image transmission over correlated channels [9], with benefits including extended battery life [11], enhanced performance, and robust diversity techniques for future networks [10]. The capacity to extract both local spectral and temporal information is the fundamental benefit of the Wavelet Transform over the Fourier Transform in OFDM based on the DWT [12]. Wavelet transform is benefit for identifying signals, de-noising, and compression due to its superior energy compaction capabilities. Scaling and translation of a short wave are used to represent a signal. This wave is sometimes referred to as a wavelet. The wavelets’ average value is consistently zero, and the interval is always bounded [13]. Wavelet families include Haar Wavelet [14], Daubechies Wavelekt (DW) [15], Symlets Wavelet (SW) [16], Coiflet Wavelet (CW) [17], Biorthogonal Wavelet (BW) [18], Reverse Biorthogonal Wavelet (RBW) [19], Discrete Meyer (DM) [20], Embedded Zerotree Wavelet (EZW) [21], Spatial Orientation Tree Wavelet (SOTW) [22], Wavelet Difference Reduction (WDR) [23]. We are interested in the Haar wavelet type [14] in this study since it is one of the most prominent in mathematics and engineering due to its simplicity and compact support. The Haar Wavelet divides a discrete signal into two equal-sized components. The first section is called the trending section, while the second section is known as the fluctuation section. In this paper, we focus on the Haar wavelet type. The authors of [3] present many ways for quickly implementing various types of wavelet transformations. Because of its strong performance in high-noise and multi-path situations, the DWT is being used more and more in wireless communication systems [24]. By improving spectrum efficiency and lowering BER, DWT-based systems can perform better in 5G than conventional DFT-based techniques, particularly in Non-Orthogonal Multiple Access (NOMA) systems [24]. In MIMO-OFDM systems, DWT is also utilized to maintain high data rates while lowering power consumption and computational complexity [24]. Furthermore, modulation and coding performance in contemporary communication channels are improved by DWT’s capacity to manage multi-resolution signal analysis [24].

## II. Related work

Recent advancements in OFDM systems have seen significant use of FDE to counteract the impacts of multipath fading. In DFT-OFDM systems, one of the key advantages is the straightforward implementation of FDE, which enhances system performance by simplifying the process of handling channel distortions. Several studies have focused on improving the efficiency of FDE-based OFDM systems. In [25] FDE-based OFDM systems with index modulation (OFDM-IM) have been explored for their ability to enhance spectral efficiency by employing subcarrier activation patterns and reducing the active subcarrier count. These innovations aim to maximize data throughput while maintaining the reliability of communication channels, making them suitable for 5G and beyond.

A recent study in [26] proposes an iterative decision feedback channel estimation (IDFCE) method for DFT-Spread OFDM (DFT-S-OFDM) systems, aimed at improving channel estimation and error correction. The method integrates a time-division multiplexing reference signal into a turbo FDE. In this approach, channel responses, which are used in both feedforward and decision-feedback equalizers, are iteratively updated during each iteration of the turbo FDE, enhancing the accuracy of the estimation process. A novel FDE scheme designed for one-bit uplink multi-user massive MIMO systems is presented in [27]. This scheme employs a pseudo-random quantization (PRQ) method with non-zero threshold quantization to mitigate the effects of quantization distortion, which typically limits the system’s ability to handle high-order modulation. The proposed equalizer, based on Newton’s method (NM), is specifically tailored for OFDM transmissions under frequency-selective fading conditions by leveraging the inherent benefits of massive MIMO. To reduce computational complexity, a low-complexity FDE algorithm is developed using a quasi-Newton approach. However, despite its effectiveness, FDE in DFT-OFDM introduces additional computational complexity due to the requirement for extra DFT and IDFT operations during the equalization process. This extra step not only increases processing time but also limits the system’s efficiency, particularly in high-speed communication scenarios where real-time performance is crucial.

The study presented in [28] focused on the multilevel redundant DWT-OFDM (ML-RDWT-OFDM) system. This system incorporates Low-Density Parity-Check (LDPC) coding and Soft Decision (SD) decoding based on the belief propagation algorithm to enhance the overall system performance. Simulation results demonstrated that incorporating LDPC coding significantly improves the performance of ML-RDWT-OFDM systems.

A comparative study analyzed the BER performance of signals transmitted using DWT and DFT in various channel environments was presented in [29]. It was observed that DWT-OFDM and DFT-OFDM perform similarly in AWGN channels, but DFT-OFDM exhibits better performance in Rayleigh channels. A recent study in [30] proposed an adaptive decision feedback equalizer with interference reconstruction and cancellation for OFDM systems. This method minimizes the symbol error rate (MSER) by performing adaptive filtering in the delay dimension, mitigating inter-symbol interference (ISI), and compensating for cyclic convolution and phase flipping.

The limitations of traditional FDE implementations have sparked interest in exploring alternative approaches, such as the use of wavelet-based OFDM systems. DWT-OFDM, for instance, offers improved spectral efficiency and better time-frequency localization compared to DFT-OFDM. However, conventional FDE implementations in DWT-OFDM still suffer from the same computational drawbacks due to the additional DFT/IDFT operations required for equalization. This has highlighted the need for more advanced equalization techniques. The proposed JLCRLZF-WDE addresses these challenges by eliminating the need for the extra DFT/IDFT steps, reducing computational complexity, and improving BER performance, making it a more efficient solution for modern wireless communication systems. The proposed equalizer can execute both equalization and co-CFO compensation operations at the same time without requiring extra blocks of DFT/IDFT. Moreover, research on DWT-OFDM is still in its early stages, and there is a lot of scope for additional performance enhancement. For the MIMO-OFDM system, this paper’s contributions are summarized below:

1. The JLCRLZF-WDE is offered as an appropriate term for a tool that performs the equalization and co-CFO compensation operations combined in the WD rather than the FD, which requires additional IDFT/DFT blocks [31].
2. The proposed JLCRLZF-WDE equalizer employs a constant value to decrease the time delay necessary for SNR estimation, as in the LMMSE equalizer. This value is known as the Regularization parameter, and it is responsible for noise amplification mitigation in the LZF equalizer.
3. The proposed JLCRLZF-WDE can conduct its process appropriately for varied values of the normalized CFO, as well as in the situation of Co-CFO.
4. The performance of the proposed JLCRLZF-WDE is determined in terms of BER and simulated time, using the LMMSE-WDE as a benchmark for comparison.
5. The obtained results indicate that the proposed JLCRLZF-WDE is resistant to estimation errors as well as different values of the normalized co-CFO.

## III. Proposed DWT-OFDM system model

**Fig 1:**
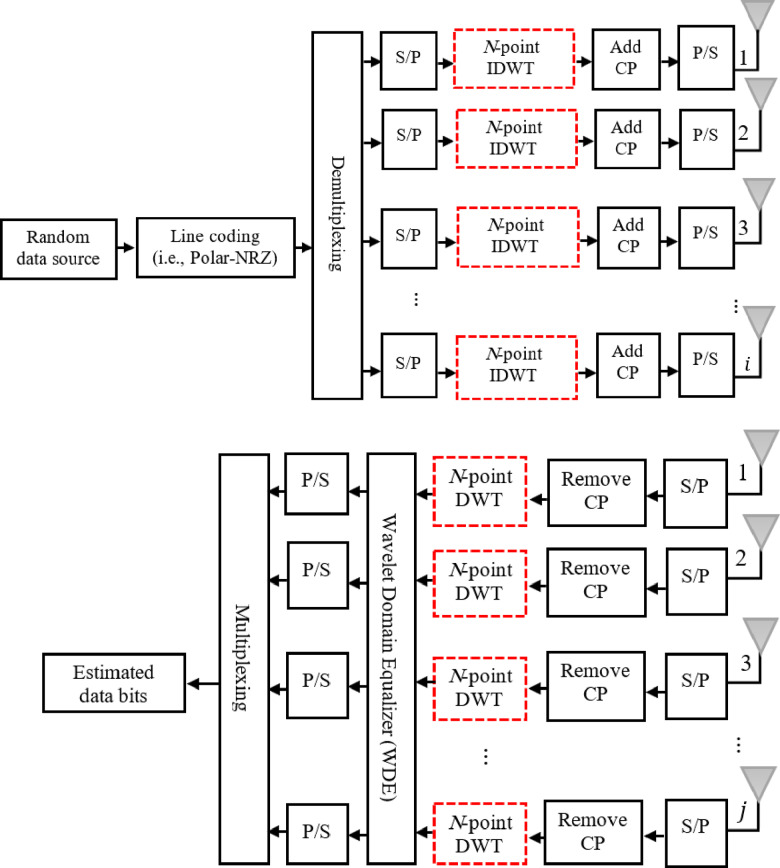
The *i* ×* j* MIMO-OFDM structure based on DWT utilizing WDE.

Fig 1 depicts the main transceiver topology of an *i × j* MIMO-DWT-OFDM system employing WDE over a Rayleigh fading channel. The proposed JLCRLZF-WDE is used to accomplish the equalization and co-CFO compensation operations in the WD.

Firstly, a polar NRZ is generated via a random that de-multiplexed via each stream in accordance to antennas number at the transmitter side. A Serial-to-Parallel (S/P) converter is used to route each group of length *N* . IDWT comes after that. As a result, the transmitted data vector before the IDWT block associated with the *i*^th^ vector may be written as:


$${\text{X}}^{i}={\left[\begin{array}{ccc} X_{0}^{i} & X_{1}^{i} & \begin{array}{ccc} \ldots .. & X_{N-2}^{i} & X_{N-1}^{i} \end{array} \end{array}\right]}^{T}$$ (1)

where ${\left(.\right)}^{T}$ is the matrix transpose, *N* is the sub-carrier number, and ${\text{X}}^{i}\in {\mathbb{R}}^{N\times 1}$. Currently, each *N* vector has the IDWT block applied to it. The IDWT block is now applied to each of the *N* vectors. As a consequence, the *i*^th^ vector’s IDWT output may be stated as follows:


$${\text{x}}^{i}=h_{t}u{\text{X}}^{i}={\left[\begin{array}{ccc} x_{0}^{i} & x_{1}^{i} & \begin{array}{ccc} \ldots .. & x_{N-2}^{i} & x_{N-1}^{i} \end{array} \end{array}\right]}^{T}$$ (2)

where ${\text{x}}^{i}\in {\mathbb{R}}^{N\times 1}$, $u\in {\mathbb{R}}^{2N\times N}$ represents the up-sampling matrix, which is defined as follows:



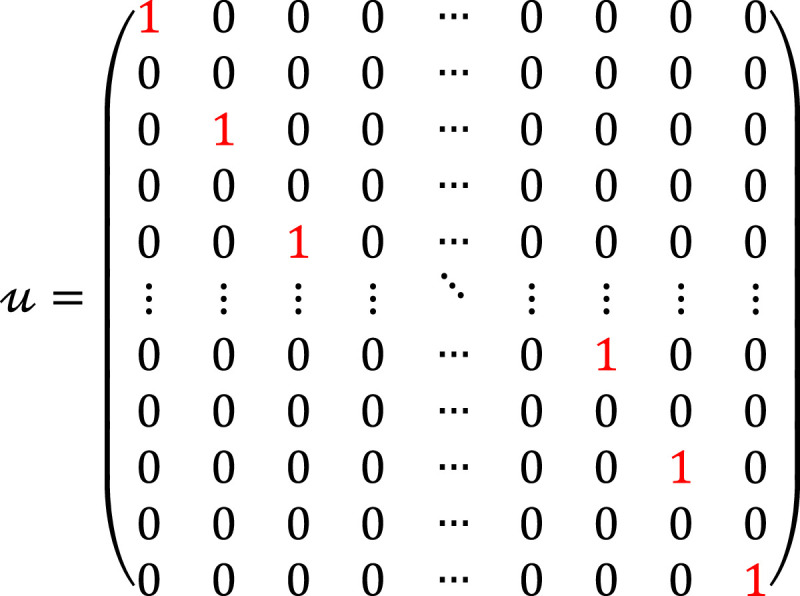

(3)


$h_{t}\in {\mathbb{R}}^{N\times 2N}$ represents the filter Impulse Response Matrix (IRM) of the IDWT at the transmitter side, which consists of the Low Pass Filter (LPF) and the High Pass Filter (HPF). It is a composite of two filters as: $h_{t}=\left[\begin{array}{ccc} h_{\text{0}} & ; & h_{1} \end{array}\right],h_{0},h_{1}\in {\mathbb{R}}^{N\times N}$, which is defined as:



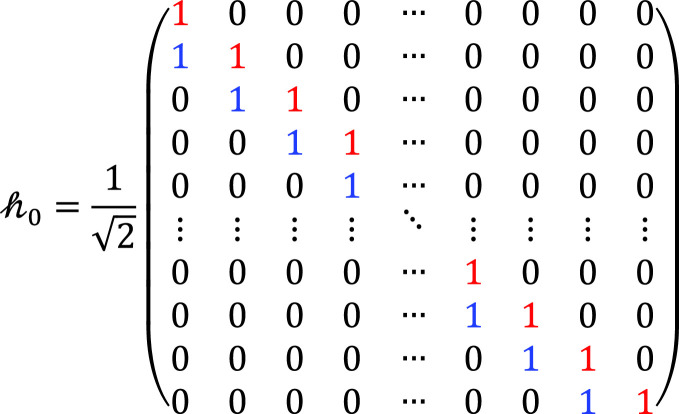

(4)


and



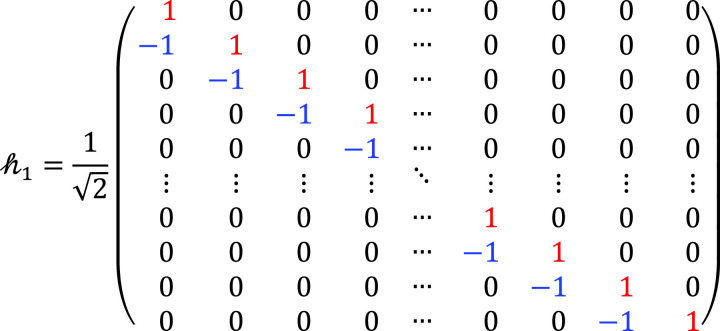

(5)


In fact, the term $h_{t}u$ matrix refers to the IDWT matrix. $h_{\text{o}}$ is the IRM of LPF at the transmitter and receiver sides and $h_{1}$ is the RM of the HPF at the transmitter side. The CP is now appended to the beginning of each vector. As a result, the *i*^th^ modulated vector with CP is written as follows:


$${\text{x}}_{\text{CP }}^{i}={\text{P}}_{{\text{CP}}^{+}}{\text{x}}^{i}={\left[\begin{array}{ccc} x_{0,\text{CP}}^{i} & x_{1,\text{CP}}^{i} & \begin{array}{ccc} \ldots .. & x_{N-2,\text{CP}}^{i} & x_{N-1,\text{CP}}^{i} \end{array} \end{array}\right]}^{T}$$ (6)

with


$${\text{P}}_{{\text{CP}}^{+}}={\left[{\left[0_{N_{\text{CP}}\times \left(N-N_{\text{CP}}\right)};\;\;I_{N_{\text{CP}}\times N_{\text{CP}}}\right]}^{T},I_{N\times N}\right]}^{T}$$ (7)

where ${\text{P}}_{{\text{CP}}^{+}}\in {\mathbb{R}}^{\left(N+N_{\text{CP}}\right)\times N}$ is the matrix related to CP insertion, ${\text{x}}_{\text{CP }}^{i}\in {\mathbb{R}}^{\left(N+N_{\text{CP}}\right)\times 1}$, $N_{\text{CP}}$represents the CP length, $I_{a\times b}$denotes an a×b diagonal unity matrix, and $0_{a\times b}$is an a×b matrix of all zero elements. The data is then sent via the Rayleigh fading channel once each stream’s Parallel-to-Serial (P/S) stage has been employed. The Rayleigh fading channel and co-CFO are included in the received data vector at the *j*^th^ antenna, and noise after the Serial-to-Parallel (S/P) stage, is represented as follows:


$${\text{y}}^{j}={\text{ψ}}^{j,i}H^{j,i}{\text{P}}_{{\text{CP}}^{+}}{\text{x}}^{i}+{\text{z}}^{j}$$ (8)

where ${\text{y}}^{j}\in {\mathbb{C}}^{\left(N+N_{\text{CP}}\right)\times 1}$ indicates the *j*^th^ vector before the ignoration of the CP vector, ${\text{z}}^{j}{\mathbb{C}}^{\left(N+N_{\text{CP}}\right)\times 1}$is the complex AWGN vector with zero mean. The co-CFO can be expressed as a diagonal matrix as ${\text{ψ}}^{j,i}\in {\mathbb{C}}^{\left(N+N_{\text{CP}}\right)\times \left(N+N_{\text{CP}}\right)}$ that given by:


$${\text{ψ}}^{j,i}\text{=Diag}\left\{1,e^{j\frac{2\pi \varepsilon_{j,i}}{N}},e^{j\frac{4\pi \varepsilon_{j,i}}{N}},\ldots ,e^{j\frac{2\pi \varepsilon_{j,i}\left(N+N_{\text{CP}}-2\right)}{N}},e^{j\frac{2\pi \varepsilon_{j,i}\left(N+N_{\text{CP}}-1\right)}{N}}\right\}$$ (9)

The co-CFO among the transmitting antenna number *i* and receiving antenna number *j* is $\varepsilon_{j,i}$ that expressed as:


$$\varepsilon_{j,i}=\frac{f}{\text{Δ}f}$$ (10)

where the sub-carrier spacing is represented by Δf and the frequency shift by *f*. Additionally, the size of transform, carrier frequency, oscillator misalignment, all have an effect on the normalized co-CFO value. The channel IRM among the *j*^th^ receiving antenna and the *i*^th^ sending antenna is denoted by $H^{j,i}\in {\mathbb{C}}^{\left(N+N_{\text{CP}}\right)\times \left(N+N_{\text{CP}}\right)}$, which is defined as:


$$H^{j,i}=\left(\begin{array}{ccccccccc} h(0) & 0 & 0 & 0 & \ldots & 0 & 0 & 0 & 0\\ h(1) & h(0) & 0 & 0 & \ldots & 0 & 0 & 0 & 0\\ \vdots & h(1) & h(0) & 0 & \ldots & 0 & 0 & 0 & 0\\ h(L-1) & \vdots & h(1) & h(0) & \ldots & 0 & 0 & 0 & 0\\ 0 & h(L-1) & \vdots & h(1) & \ldots & 0 & 0 & 0 & 0\\ 0 & 0 & h(L-1) & \vdots & \ldots & 0 & 0 & 0 & 0\\ 0 & 0 & 0 & h(L-1) & \ldots & h(0) & 0 & 0 & 0\\ 0 & 0 & 0 & 0 & \ddots & h(1) & h(0) & 0 & 0\\ 0 & 0 & 0 & 0 & \ldots & h(2) & h(1) & h(0) & 0\\ 0 & 0 & 0 & 0 & \ldots & h(3) & h(2) & h(1) & h(0) \end{array}\right)$$ (11)

The channel impulse response coefficient is represented by $h\left(w\right)$, while the number of Rayleigh fading channel taps is indicated by $w\in \left\{1,2,\ldots ,L-1\right\}$. Such ignores the head of each data vector that represents the CP. Additionally, the output of *j*^th^ vector’s CP removal stage is written as:


$${\bar{y}}^{j}={\text{P}}_{{\text{CP}}^{-}}{\text{y}}^{j}={\text{P}}_{{\text{CP}}^{-}}{\text{Λ}}^{j,i}{\text{P}}_{{\text{CP}}^{+}}{\text{x}}^{i}+{\text{P}}_{{\text{CP}}^{-}}{\text{z}}^{j}$$ (12)

where ${\text{Λ}}^{j,i}={\text{ψ}}^{j,i}H^{j,i}$denotes a composite of both the co-CFO and Rayleigh fading channel. Meanwhile, ${\text{P}}_{{\text{CP}}^{-}}\in {\mathbb{R}}^{N\times \left(N+N_{\text{CP}}\right)}$ denotes the CP removal matrix. The above equation can be expressed using Eq. (2) as follows:


$$\left[\begin{array}{c} {\bar{y}}^{1}\\ {\bar{y}}^{2}\\ \vdots \\ {\bar{y}}^{j} \end{array}\right]=\left[\begin{array}{cccc} {\text{P}}_{{\text{CP}}^{-}}\wedge^{1,1}{\text{P}}_{{\text{CP}}^{+}}h_{t}u & {\text{P}}_{{\text{CP}}^{-}}\wedge^{1,2}{\text{P}}_{{\text{CP}}^{+}}h_{t}u & \ldots & {\text{P}}_{{\text{CP}}^{-}}\wedge^{1,i}{\text{P}}_{{\text{CP}}^{+}}h_{t}u\\ {\text{P}}_{{\text{CP}}^{-}}\wedge^{2,1}{\text{P}}_{{\text{CP}}^{+}}h_{t}u & {\text{P}}_{{\text{CP}}^{-}}\wedge^{2,2}{\text{P}}_{{\text{CP}}^{+}}h_{t}u & \ldots & {\text{P}}_{{\text{CP}}^{-}}\wedge^{2,i}{\text{P}}_{{\text{CP}}^{+}}h_{t}u\\ \vdots & \vdots & \ddots & \vdots \\ {\text{P}}_{{\text{CP}}^{-}}\wedge^{j,1}{\text{P}}_{{\text{CP}}^{+}}h_{t}u & {\text{P}}_{{\text{CP}}^{-}}\wedge^{j,2}{\text{P}}_{{\text{CP}}^{+}}h_{t}u & \ldots & {\text{P}}_{{\text{CP}}^{-}}\wedge^{j,i}{\text{P}}_{{\text{CP}}^{+}}h_{t}u \end{array}\right]\left[\begin{array}{c} {\text{X}}^{1}\\ {\text{X}}^{2}\\ \vdots \\ {\text{X}}^{i} \end{array}\right]+\left[\begin{array}{c} {\text{P}}_{{\text{CP}}^{-}}{\text{z}}^{1}\\ {\text{P}}_{{\text{CP}}^{-}}{\text{z}}^{2}\\ \vdots \\ {\text{P}}_{{\text{CP}}^{-}}{\text{z}}^{j} \end{array}\right]$$ (13)

Then, an *N*-point DWT is applied, comprising two key operations: combination of LPF and HPF $h_{r}$ and the down-conversion matrix *d*. Hence, $h_{r}\in {\mathbb{R}}^{2N\times N}$, $h_{r}=\left[h_{0};h_{2}\right],h_{0},h_{2}\in {\mathbb{R}}^{N\times N}$, where



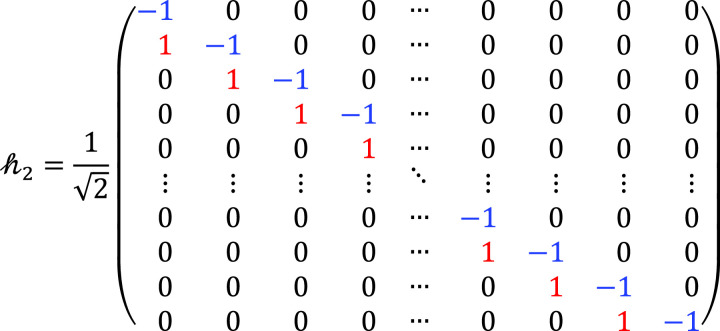

(14)


where $h_{2}$ is the RM of the HPF at the receiver side. Now, the down-sampling matrix $d\in {\mathbb{R}}^{N\times 2N}$ is applied, which is defined as follows:



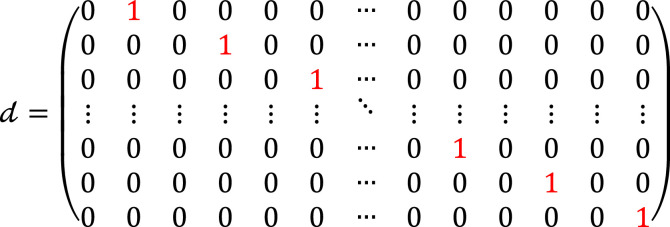

(15)


Now, Eq. (13) will be modified to include DWT as follows:


$$\left[\begin{array}{c} {\tilde{y}}^{1}\\ {\tilde{y}}^{2}\\ \vdots \\ {\tilde{y}}^{j} \end{array}\right]=\left[\begin{array}{cccc} dh_{r}{\text{P}}_{{\text{CP}}^{-}}{\text{Λ}}^{1,1}{\text{P}}_{{\text{CP}}^{+}}h_{t}u & dh_{r}{\text{P}}_{{\text{CP}}^{-}}{\text{Λ}}^{1,2}{\text{P}}_{{\text{CP}}^{+}}h_{t}u & \ldots & dh_{r}{\text{P}}_{{\text{CP}}^{-}}{\text{Λ}}^{1,i}{\text{P}}_{{\text{CP}}^{+}}h_{t}u\\ dh_{r}{\text{P}}_{{\text{CP}}^{-}}{\text{Λ}}^{2,1}{\text{P}}_{{\text{CP}}^{+}}h_{t}u & dh_{r}{\text{P}}_{{\text{CP}}^{-}}{\text{Λ}}^{2,2}{\text{P}}_{{\text{CP}}^{+}}h_{t}u & \ldots & dh_{r}{\text{P}}_{{\text{CP}}^{-}}{\text{Λ}}^{2,i}{\text{P}}_{{\text{CP}}^{+}}h_{t}u\\ \vdots & \vdots & \ddots & \vdots \\ dh_{r}{\text{P}}_{{\text{CP}}^{-}}{\text{Λ}}^{j,1}{\text{P}}_{{\text{CP}}^{+}}h_{t}u & dh_{r}{\text{P}}_{{\text{CP}}^{-}}{\text{Λ}}^{j,2}{\text{P}}_{{\text{CP}}^{+}}h_{t}u & \ldots & dh_{r}{\text{P}}_{{\text{CP}}^{-}}{\text{Λ}}^{j,i}{\text{P}}_{{\text{CP}}^{+}}h_{t}u \end{array}\right]\left[\begin{array}{c} {\text{X}}^{1}\\ {\text{X}}^{2}\\ \vdots \\ {\text{X}}^{i} \end{array}\right]+\left[\begin{array}{c} dh_{r}{\text{P}}_{{\text{CP}}^{-}}{\text{z}}^{1}\\ dh_{r}{\text{P}}_{{\text{CP}}^{-}}{\text{z}}^{2}\\ \vdots \\ dh_{r}{\text{P}}_{{\text{CP}}^{-}}{\text{z}}^{j} \end{array}\right]$$ (16)

## IV. Proposed JLCRLZF-WDE

This part discuss the mathematical representation of the proposed JLCRLZF-WDE. In the case of 2 × 2 MIMO-DWT-OFDM system, Eq. (16) will be re-written as:


$$\left[\begin{array}{c} {\tilde{y}}^{1}\\ {\tilde{y}}^{2} \end{array}\right]=\left[\begin{array}{cc} dh_{r}{\text{P}}_{{\text{CP}}^{-}}{\text{Λ}}^{1,1}{\text{P}}_{{\text{CP}}^{+}}h_{t}u & dh_{r}{\text{P}}_{{\text{CP}}^{-}}{\text{Λ}}^{1,2}{\text{P}}_{{\text{CP}}^{+}}h_{t}u\\ dh_{r}{\text{P}}_{{\text{CP}}^{-}}{\text{Λ}}^{2,1}{\text{P}}_{{\text{CP}}^{+}}h_{t}u & dh_{r}{\text{P}}_{{\text{CP}}^{-}}{\text{Λ}}^{2,2}{\text{P}}_{{\text{CP}}^{+}}h_{t}u \end{array}\right]\left[\begin{array}{c} {\text{X}}^{1}\\ {\text{X}}^{2} \end{array}\right]+\left[\begin{array}{c} dh_{r}{\text{P}}_{{\text{CP}}^{-}}{\text{z}}^{1}\\ dh_{r}{\text{P}}_{{\text{CP}}^{-}}{\text{z}}^{2} \end{array}\right]$$ (17)

Let’s define $\Pi^{j,i}\in {\mathbb{C}}^{N\times N},\Pi^{j,i}=dh_{r}{\text{P}}_{{\text{CP}}^{-}}{\text{Λ}}^{j,i}{\text{P}}_{{\text{CP}}^{+}}h_{t}u$, $j,i=\left\{1,2\right\}$. In general, $\Pi^{j,i}$ is a composite transmission matrix, representing the signal transformation up to the point of equalization and excluding the equalization process between the *i*^th^ transmitting antennas, and *j*^th^ receiving antenna. Now, Eq. (17) will be re-written as:


$$\left[\begin{array}{c} {\tilde{y}}^{1}\\ {\tilde{y}}^{2} \end{array}\right]=\left[\begin{array}{cc} \Pi^{1,1} & \Pi^{1,2}\\ \Pi^{2,1} & \Pi^{2,2} \end{array}\right]\left[\begin{array}{c} {\text{X}}^{1}\\ {\text{X}}^{2} \end{array}\right]+\left[\begin{array}{c} \phi^{1}\\ \phi^{2} \end{array}\right]$$ (18)

where $\phi^{j}=dh_{r}{\text{P}}_{{\text{CP}}^{-}}{\text{z}}^{j}$, $j=\left\{1,2\right\}$. $\phi^{j}$ is the processed noise vector, which corresponds to the noise vector after applying processing steps like CP removal, down-sampling, and filtering (e.g., LPF and HPF). 2a illustrates the magnitude of the Interference Matrix (IM) in the case of DWT, providing insight into the distribution of interference among subcarriers. In contrast, 2b depicts the corresponding phase angles of each element in 2a, offering a comprehensive view of the IM’s complex structure. The diagonal elements in 2a represent the desired subcarriers, which are free of interference, while the off-diagonal elements correspond to interference contributions from other subcarriers. To further clarify, 3a focuses on a specific instance by presenting the magnitude of the IM for row 20, enabling a more detailed examination of the interference pattern. Additionally, 3b complements this by illustrating the corresponding phase angles for the same row, as shown in 3a, thereby highlighting the interplay between magnitude and phase in the IM structure. This detailed visualization underscores the characteristics of interference in the system and aids in understanding its impact on the overall performance.

**Fig 2:**
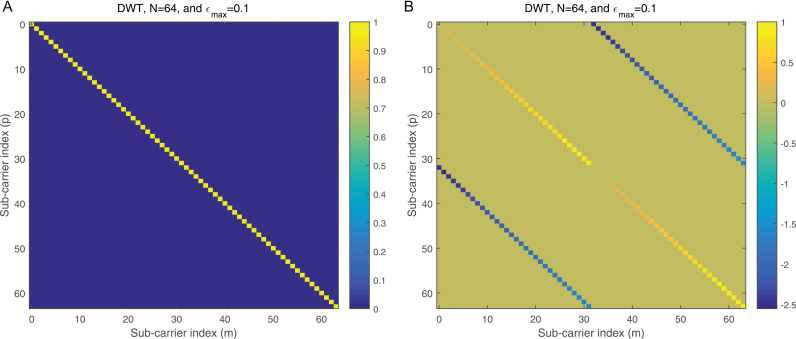
a: The IM magnitude vs the sub-carrier indices in the case of DWT. b: The IM angle vs the sub-carrier indices in the case of DWT.

**Fig 3:**
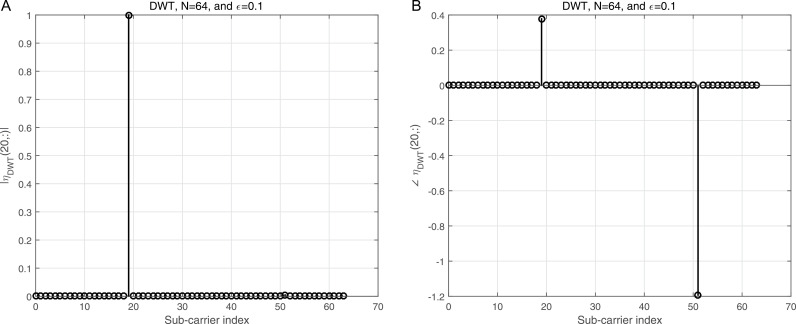
a: The IM magnitude vs the sub-carrier indices using DWT for row number 20 of Fig. 2a. b: The IM angle vs the sub-carrier indices using DWT for row number 20 of Fig 2b.

**Fig 4:**
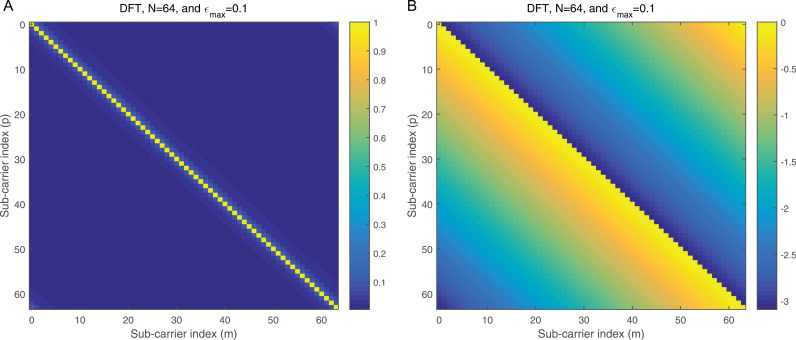
a: The IM magnitude vs the sub-carrier indices using DFT. b: The IM angle vs the sub-carrier indices using DFT.

**Fig 5:**
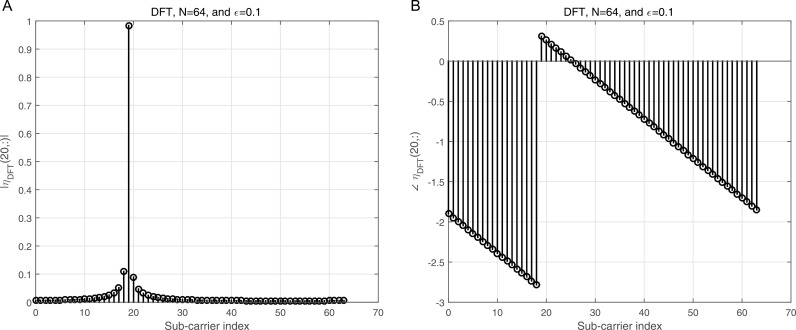
a: The IM magnitude vs the sub-carrier indices using DFT for row number 20 of Fig 4a. b: The IM angle vs the sub-carrier indices using DFT for row number 20 of Fig 4b.


Similarly, Fig 4a illustrates the magnitude of the IM for the case of DFT, providing a detailed representation of interference across subcarriers. Correspondingly, Fig 4b shows the phase angles for each element in Fig 4a, offering a comprehensive understanding of both magnitude and phase in the DFT-based IM. For a more focused analysis, Fig 5a presents the magnitude of the DFT IM for row 20, isolating the interference pattern for this specific subcarrier. In parallel, Fig 5b displays the corresponding phase angles for the same row, as shown in Fig 5a, enabling a deeper examination of the IM’s characteristics. When compared to the results shown in Fig 2, and Fig 3, it is evident that the DWT approach exhibits significantly lower interference from neighboring subcarriers to the desired subcarriers, as reflected in the diagonal elements of the IM. This demonstrates the superior ability of the DWT to mitigate interference when contrasted with the DFT, especially in systems experiencing co-CFO effects. The IM for the case of DFT under co-CFO conditions can be mathematically expressed as [32]

$${\left(\eta^{m,p}\right)}_{\text{DFT}}=e^{j\frac{\pi \left[\left(m-p\right)+\varepsilon \right]\left(N-1\right)}{N}}.\frac{\sin\left(\pi \left[\left(m-p\right)+\varepsilon_{j,i}\right]\right)}{N.\sin\left(\frac{\pi \left[\left(m-p\right)+\varepsilon_{j,i}\right]}{N}\right)}$$ (19)

The IM in the case of DWT with co-CFO is written as:


$${\left(\eta^{m,p}\right)}_{\text{DWT}}=dh_{r}{\text{ψ}}^{j,i}h_{t}u$$ (20)

So, we can takthe number of sub-carriers into consideration and ignore the remaining as:


$${\left(\mu^{j,i}\right)}_{m,p}= \{\begin{array}{c} {\left(\Pi^{j,i}\right)}_{m,p}\left|m-p\right|\leq \tau \\ 0\left|m-p\right|>\tau \end{array}$$ (21)

where m−pis the relative sub-carrier distance, and $\mu^{j,i}$is the BMA of the $\Pi^{j,i}$ matrix.

The proposed JLCRLZF-WDE general matrix solution is:


$$\Upsilon_{\text{JLCRLZF}-\text{WDE}}={\left(\mu^{H}\mu +\alpha I_{jN\times jN}\right)}^{-1}\mu^{H}$$ (22)

In Eq. (22), α represents the regularization parameter, which is a constant value used to mitigate noise amplification, and $\mu \in {\mathbb{C}}^{iN\times jN}$ is the BMA. In the case of 2×2MIMO configuration, we have:


$$\mu =\left[\begin{array}{cc} \mu^{1,1} & \mu^{1,2}\\ \mu^{2,1} & \mu^{2,2} \end{array}\right]$$ (23)

Using the JCLRLZF-WDE matrix solution stated in Eq. (19) and the BMA defined in Eq. (20). The optimal value of α, which correlates to the inverse value of the SNR, is required for the proposed equalizer. The BMA bandwidth (τ) must be accurately provided in order to satisfy reduced complexity implementation.

## V. Simulation results and analysis

The simulation results for the proposed JLCRLZF-WDE based on DWT-OFDM systems will be analyzed. The analysis will utilized a 6-tap Rayleigh fading with carrier frequency 2 GHz, modeled according to the Jakes model [33,34] and vehicle A model [35] using the simulation parameters indicated in Table 1. According to the preceding section’s analysis, the value of α should be specified with minimal BER performance and τ should be as small as feasible to reduce computational complexity.

**Table 1:** List of used parameters.

Parameter	Value	Parameter	Value
IDWT/DWT size	64	SNR range	0:5:30
IDFT/DFT size	64	Number of Tx. antennas	2
CP length	16	Number of Rx. antennas	2
Channel type	Rayleigh	Simulation model	Monte Carlo
ε_max_	0.1	Number of iterations	10^3^
Channel estimation type	Perfect		

**Fig 6:**
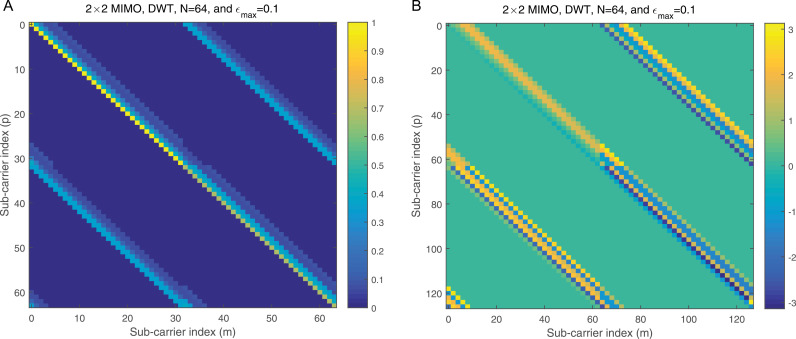
a: The normalized magnitude against the sub-carrier indices of the$\Pi^{1,1}$matrix in the case of DWT. b: The normalized angle against the sub-carrier indices of thez$\Pi^{1,1}$matrix in the case of DWT.

**Fig 7:**
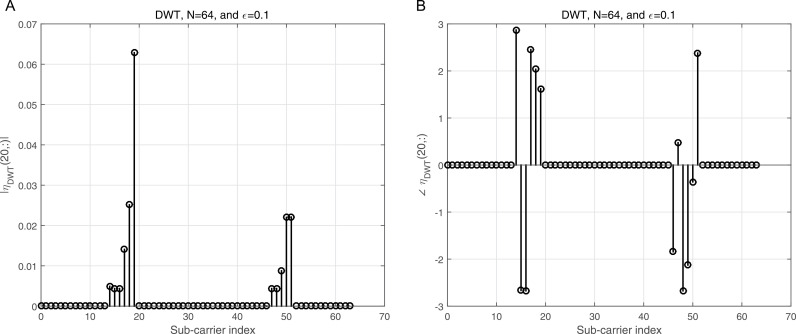
a: The normalized magnitude vs the sub-carrier indices for row number 20 of the$\Pi^{1,1}$matrix in the case of DWT illustrated in Fig 6a. b: The normalized angle vs the sub-carrier indices for row number 20 of the$\Pi^{1,1}$matrix in the case of DWT illustrated in Fig 6b.

Fig 6a shows the normalized magnitude plotted against the indices for the $\Pi^{1,1}$ matrix in the case of DWT, providing a clear visualization of how the interference distribution varies across subcarriers. Fig 6b complements this by presenting the corresponding phase angles for each element in Fig 6a, offering a comprehensive representation of both magnitude and phase characteristics. In this scenario, each normalized co-CFO is treated as a random variable with a uniform distribution in the range $\left[-\varepsilon_{max},+\varepsilon_{max}\right]$, where $\varepsilon_{max}$ denotes the maximum normalized co-CFO. This assumption captures the variability introduced by different co-CFO values in practical systems. To provide additional clarity, Fig 7a focuses on row 20 of the $\Pi^{1,1}$ matrix shown in Fig 6a, illustrating the normalized amplitude for this specific row. Meanwhile, Fig 7b presents the corresponding phase angles for the same row, as depicted in Fig 7a. These figures together offer a detailed insight into the behavior of the matrix for individual subcarriers, facilitating a deeper understanding of the DWT’s response to co-CFOs.

**Fig 8:**
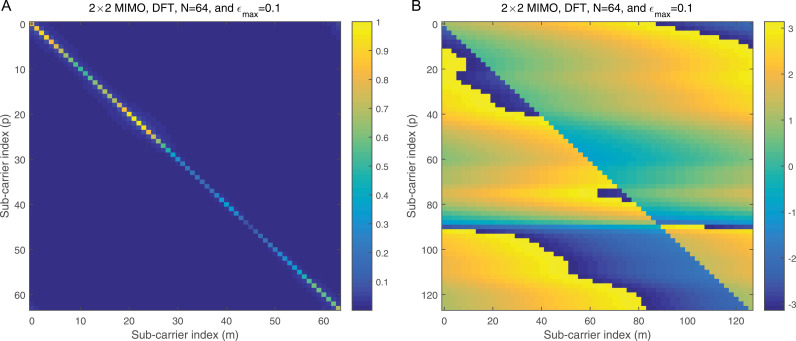
a: The normalized magnitude vs the sub-carrier indices of the$\Pi^{1,1}$matrix in the case of DFT. b: The normalized angle vs the sub-carrier indices of the $\Pi^{1,1}$matrix in the case of DFT.

**Fig 9:**
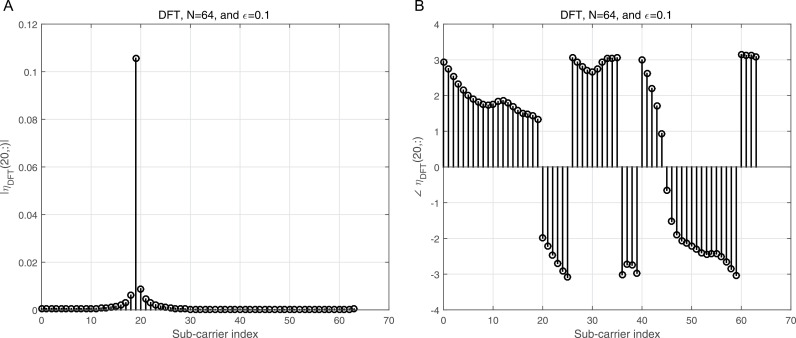
a: The normalized magnitude vs the sub-carrier indices for row number 20 of the$\Pi^{1,1}$. matrix in the case of DFT illustrated in Fig 8a. b: The normalized angle vs the sub-carrier indices for row number 20 of the$\Pi^{1,1}$. matrix in the case of DFT illustrated in Fig 8b.

Fig 8a presents the normalized magnitude plotted against the indices for the $\Pi^{1,1}$ matrix in the context of the DFT. This graphical representation provides a clear visualization of how the magnitude varies across the elements of the matrix. In contrast, Fig 8b illustrates the corresponding angles for each of the elements shown in Fig 8a, offering insights into the phase relationships within the matrix. From these figures, it becomes evident that the BMA approach is effective when applied to DFT, as it shows a clear and interpretable structure in both magnitude and phase. However, when considering the case of the DWT, the BMA appears to be less effective, which is something that will be further explored in the subsequent simulations. To provide additional clarity, Fig 9a zooms in on row number 20 from Fig 8a, presenting its normalized amplitude specifically. Fig 9b, in turn, displays the corresponding angle values for this row, offering a more detailed understanding of the phase information for a particular subset of the matrix. The analysis in these figures highlights the differences in how the BMA handles DFT versus DWT, pointing to the need for alternative methods in wavelet-based analysis. This will be examined in greater detail in the following sections.

**Fig 10:**
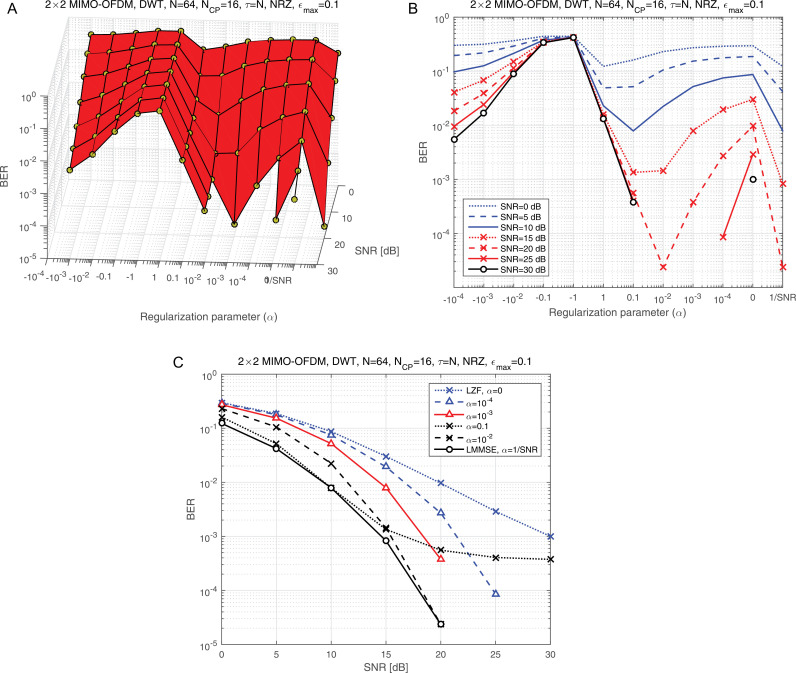
a: The BER against α at different SNR values. b: The elevation view of panel a. c: The side view of panel a.

Fig 10a illustrates the BER performance as a function of the regularization parameter (α) for various SNR values, providing a detailed analysis of how (α) impacts system performance. To offer a different perspective, Fig 10b presents an elevation view of Fig 10a, specifically for$\varepsilon_{max}=0.1$ allowing for a more nuanced examination of the parameter’s behavior. The LMMSE equalizer, as a benchmark, is represented by the final vector value of α=1/SNR, which helps to contextualize the proposed adjustments. From Fig 10b, it is clear that the best BER performance occurs at specific values of α, namely 10^-2^, 10^-3^, and 10^-4^. This indicates that selecting an optimal α is critical for achieving minimal BER. To determine the most effective value of α for subsequent simulations, the BER performance across SNR values must be thoroughly evaluated at these candidate levels. Fig 10c provides further insights by plotting BER versus SNR for different α values. The results indicate that α=10^-2^ consistently delivers optimal performance across a range of SNR conditions, particularly when used with LMMSE equalization. This robustness makes α=10^-2^ the ideal choice for the remaining simulations, ensuring stable and reliable system performance under varying conditions. Moving forward, our primary focus shifts to evaluating the BMA parameter (τ) with the aim of simplifying the equalization design. This analysis will contribute to a more efficient and practical implementation of the equalization process, potentially enhancing overall system usability.

**Fig 11:**
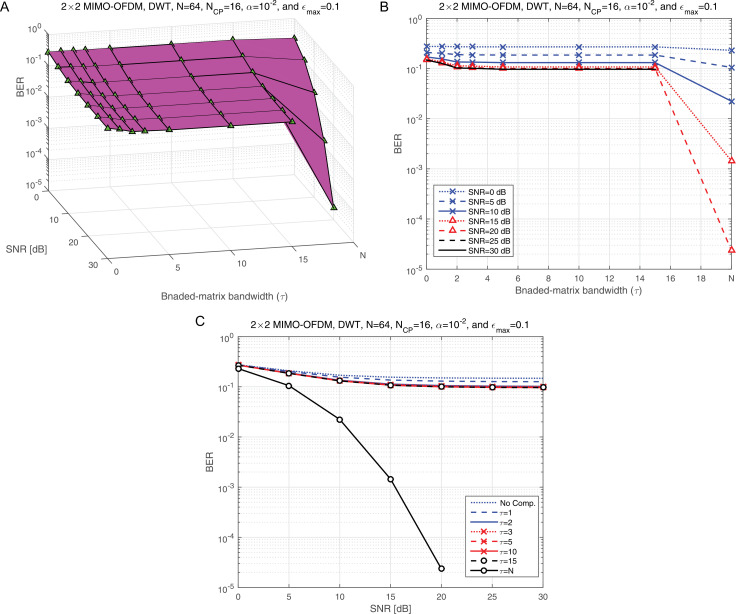
a: The BER against SNR at different compensation scenarios. b: The elevation view of panel a. c: The side view of panel a.

Fig 11a illustrates the BER performance as a function of the BMA parameter (τ) for various SNR levels, highlighting the relationship between τ and system reliability. Fig 11b provides an elevation view of Fig 11a, offering a three-dimensional perspective that helps visualize how τ interacts with SNR to influence BER. This dual representation enhances the understanding of the trade-offs associated with different τ settings. The last two vector values in Fig 11a and 11b correspond to two critical scenarios: the BMA condition (τ= 15) and the full compensation scenario (τ= *N*, where *N* represents the total number of subcarriers). A clear disparity is observed in BER performance between these scenarios, with τ= *N* significantly outperforming τ=15. This difference underscores the importance of selecting an optimal τ value for maintaining low BER and achieving robust system performance. For subsequent simulations, the full compensation scenario (τ= *N*) will be employed, as it consistently demonstrates superior BER performance under varying conditions. This decision is further validated by Fig 11c, which presents BER performance as a function of SNR across different compensation scenarios. The results clearly show that robust BER performance is only achieved under the full compensation condition, where τ=N. This analysis emphasizes the critical role of *τ* in system design. While partial compensation (e.g., τ=15) offers computational simplicity, it introduces a significant degradation in BER performance. In contrast, the full compensation scenario ensures maximum reliability, making it the optimal choice for high-performance applications. This finding will guide the remaining simulations and provide a foundation for future investigations into the trade-offs between complexity and performance in compensation mechanisms.

**Fig 12:**
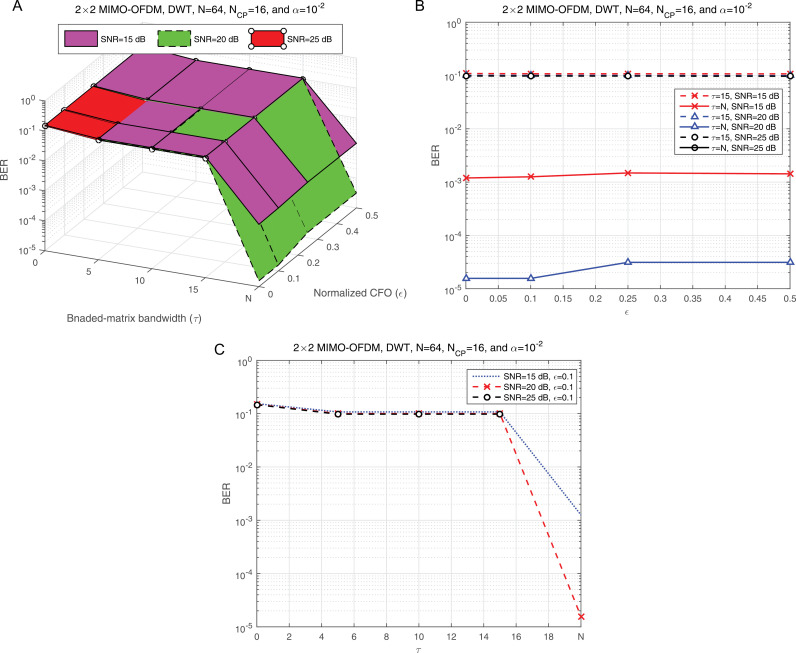
a: The BER at different values of the compensation scenarios and CFO. b: The elevation view of panel a. c: The side view of panel a.

Fig 12a illustrates the BER performance of the proposed JLCRLZF-WDE as a function of the normalized co-CFO (ε) at various BMA levels (τ). This representation highlights the impact of different compensation levels on the system’s ability to mitigate co-CFO effects. To provide a more comprehensive perspective, Fig 12b presents an elevation view of Fig 12a, focusing on the performance under two key compensation scenarios: partial compensation (τ=15) and full compensation (τ=N). At a normalized co-CFO value of ε = 0.1, Fig 12c offers a side view of Fig 12a, further emphasizing the differences in performance between these scenarios. These visualizations collectively reveal that the proposed JLCRLZF-WDE achieves reliable BER performance only when full compensation (τ=N) is employed. Partial compensation (τ=15) results in a noticeable degradation, indicating that insufficient compensation that cannot adequately handle the interference caused by co-CFOs.

This analysis highlights the critical interplay between the compensation strategy (τ) and the robustness of the JLCRLZF-WDE. While partial compensation may reduce computational complexity, it fails to provide the level of interference mitigation necessary for acceptable performance, especially in scenarios with significant co-CFO effects. Conversely, full compensation ensures that the JLCRLZF-WDE effectively minimizes interference and achieves optimal BER performance across the tested range of normalized co-CFO values. Based on these findings, the following simulations will adopt a full compensation strategy (τ=N), paired with a regularization parameter of α = 10^ − 2^. This configuration reflects the optimal balance between interference mitigation and algorithmic robustness, ensuring that the proposed JLCRLZF-WDE performs reliably under various operating conditions. These insights also pave the way for future studies into the trade-offs between computational complexity and performance in advanced equalization techniques.

**Fig 13:**
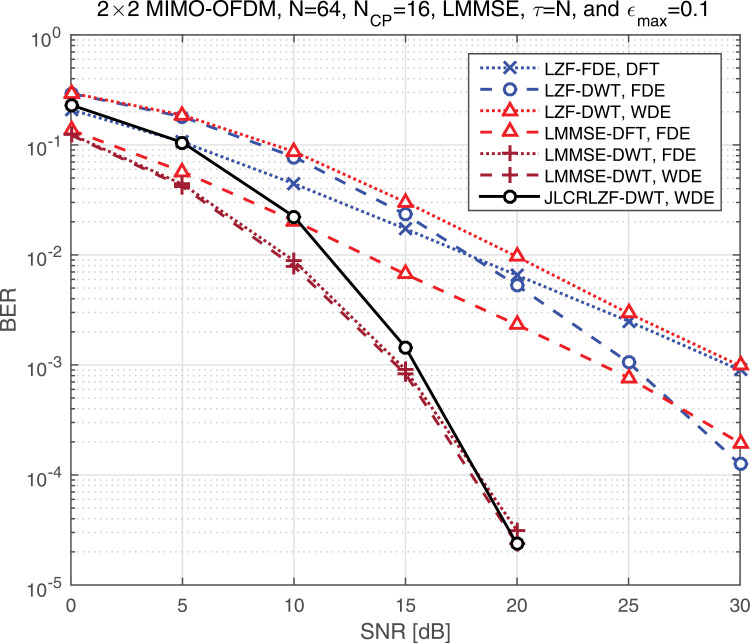
The BER vs the SNR for the considered schemes.

Fig 13 presents the BER performance as a function of SNR for various linear equalizers operating in both the Frequency Domain (FD) and the Wavelet Domain (WD), providing a comprehensive comparison of their efficiency under different scenarios. The analysis in Table 2 uses the LMMSE-WDE as the reference benchmark, which is widely recognized for its robust performance in mitigating interference and achieving low BER. A closer examination reveals significant differences in the additional SNR required by each equalizer to match the BER performance of the LMMSE-WDE at a target BER of 10^ − 3^. Specifically:

**Table 2:** The SNR difference of different equalizers of Fig 4 at BER = 10^-3^.

Equalizer type	Transform type	FDE	WDE	SNR [dB]	Diff. [dB]
**LMMSE**	DWT		✓	14.58	0.00
**LZF**	DWT		✓	29.93	15.35
**LZF**	DWT	✓		25.12	10.54
**LMMSE**	DWT	✓		14.79	0.21
**LZF**	DFT	✓		29.47	14.89
**LMMSE**	DFT	✓		23.80	9.22
**JLCRLZF**	DWT		✓	15.40	0.82

1. LZF-WDE: This equalizer requires approximately 15.35 dB of additional SNR, indicating a substantial performance gap when compared to the LMMSE-WDE. This large difference highlights the limitations of the LZF-WDE in managing interference effectively, particularly under challenging SNR conditions.
2. LZF-FDE and LMMSE-FDE (DWT): The LZF-FDE requires an additional 10.54 dB, while the LMMSE-FDE based on the DWT only requires an additional 0.21 dB. This demonstrates that while both equalizers benefit from operating in the wavelet domain, the LMMSE-FDE performs significantly closer to the LMMSE-WDE, showcasing the advantages of combining DWT with an LMMSE approach in improving robustness.
3. LZF-FDE and LMMSE-FDE (DFT): These equalizers perform less effectively than their wavelet-based counterparts. The LZF-FDE requires an additional 14.89 dB, and the LMMSE-FDE needs 9.22 dB of extra SNR to match the LMMSE-WDE. This substantial gap emphasizes the inherent limitations of DFT-based equalization in handling interference and noise compared to DWT-based techniques.
4. Proposed JLCRLZF-WDE: Notably, the proposed JLCRLZF-WDE demonstrates remarkable performance, requiring only 0.82 dB of additional SNR to achieve the same BER as the LMMSE-WDE. This minor difference underscores the effectiveness of the JLCRLZF-WDE in leveraging advanced design principles to enhance performance, making it a strong contender for practical applications.

This analysis underscores the critical role of domain choice (DWT vs. DFT) and equalization strategy (LZF vs. LMMSE) in determining system performance. The wavelet domain provides significant advantages, as evidenced by the closer alignment of DWT-based equalizers to the LMMSE-WDE benchmark. Among all the tested equalizers, the proposed JLCRLZF-WDE emerges as a highly efficient solution, offering near-parity performance with the LMMSE-WDE while maintaining a balance between computational complexity and robustness. This finding highlights the potential of JLCRLZF-WDE for advanced communication systems, particularly in environments where interference and noise present significant challenges.

Table 2 offers a comparative analysis of the SNR requirements for achieving a BER of 10^-3^ using different equalizer types across the DWT and DFT transform domains, with both FDE and WDE approaches. The insights derived from this table reveal significant performance differences based on the choice of equalizer, transform type, and compensation strategy.

**Key Observations**

1. Reference Equalizer (LMMSE-WDE, DWT):
  - The LMMSE-WDE in the DWT domain serves as the performance benchmark with the lowest SNR requirement (14.58 dB) and a difference of 0 dB. This highlights its effectiveness as an optimal equalizer with robust interference mitigation.
2. LZF-WDE (DWT):
  - The LZF-WDE requires 29.93 dB, which is 15.35 dB higher than the LMMSE-WDE. This substantial gap underscores the LZF-WDE’s limitations in handling interference and noise in the wavelet domain compared to the LMMSE-WDE.
3. LZF-FDE (DWT):
  - It requires 25.12 dB, this configuration reduces the gap to 10.54 dB relative to the benchmark. While still inferior to the LMMSE-WDE, it demonstrates improved performance compared to the LZF-WDE due to the frequency domain equalization strategy.
4. LMMSE-FDE (DWT):
  - With a minimal additional SNR requirement of 0.21 dB (14.79 dB total), this equalizer achieves near-parity with the LMMSE-WDE. This showcases the LMMSE-FDE’s ability to maintain robust performance in the DWT domain with only a slight compromise in efficiency.
5. Equalizers in the DFT domain:
  - Both the LZF-FDE and LMMSE-FDE show higher SNR requirements compared to their DWT counterparts:
    - LZF-FDE (DFT): It requires 29.47 dB, 14.89 dB higher than the benchmark.
    - LMMSE-FDE (DFT): It requires 23.80 dB, 9.22 dB above the benchmark.
  - These results emphasize the limitations of DFT-based systems in achieving the same level of interference suppression and noise resilience as DWT-based systems.
6. Proposed JLCRLZF-WDE (DWT):
  - The proposed equalizer achieves a strong balance between performance and efficiency, requiring only 15.40 dB, which is just 0.82 dB higher than the LMMSE-WDE. This minor difference highlights its ability to effectively handle interference while operating close to optimal conditions.

Thus, Table 2 demonstrates the clear advantages of the DWT over the DFT for both FDE and WDE approaches. Equalizers operating in the DWT domain consistently require lower SNR values to achieve the target BER of 10^ − 3^, showcasing the wavelet transform’s superior ability to localize interference and noise in time and frequency. The LMMSE equalizers outperform the LZF counterparts across all configurations, highlighting the effectiveness of LMMSE in leveraging optimal weighting strategies for interference mitigation. Among all configurations, the proposed JLCRLZF-WDE stands out as a near-optimal solution, offering performance that closely matches the LMMSE-WDE while maintaining design simplicity and computational efficiency. This analysis underscores the critical role of choosing the appropriate domain (DWT vs. DFT) and equalizer type to optimize system performance. The findings also validate the use of the proposed JLCRLZF-WDE as a practical and efficient solution for scenarios requiring robust BER performance with minimal additional SNR.

Additionally, as Table 3 provides critical insights into the performance of various equalizers in achieving a BER of 10^−4^. The LMMSE-WDE in the DWT domain stands out as the optimal benchmark, requiring the least SNR (17.97 dB). This demonstrates its ability to effectively suppress interference and manage noise in high-precision communication scenarios. Among the other equalizers in the DWT domain, the LMMSE-FDE and the proposed JLCRLZF-WDE perform remarkably well, with SNR requirements of 18.28 dB and 18.24 dB, respectively. Their minimal SNR differences (0.31 dB and 0.27 dB) from the benchmark highlight their potential as robust alternatives, especially the JLCRLZF-WDE, which balances performance and simplicity.

**Table 3:** The SNR difference of different equalizers of Fig 4 at BER = 10^-4^.

Equalizer type	Transform type	FDE	WDE	SNR [dB]	Diff. [dB]
**LMMSE**	DWT		✓	17.97	0.00
**LZF**	DWT		✓	>30	>12.03
**LZF**	DWT	✓		>30	>12.03
**LMMSE**	DWT	✓		18.28	0.31
**LZF**	DFT	✓		>30	>12.03
**LMMSE**	DFT	✓		>30	>12.03
**JLCRLZF**	DWT		✓	18.24	0.27

In contrast, the LZF-based equalizers (LZF-WDE and LZF-FDE) and all DFT-based equalizers exhibit significantly poorer performance, requiring SNR values exceeding 30 dB. This large disparity underscores the limitations of both the LZF strategy and the DFT transform in achieving low BER levels, particularly in scenarios demanding high precision. These results strongly advocate for the use of DWT-based equalizers, as they consistently deliver better performance due to the wavelet domain’s superior time-frequency resolution and its ability to localize interference effectively.

Overall, the proposed JLCRLZF-WDE emerges as a practical and efficient solution, offering near-optimal performance with a minimal SNR penalty compared to the LMMSE-WDE. This makes it a compelling choice for low-BER communication systems that require high robustness with manageable computational complexity. The findings further emphasize the importance of selecting appropriate equalization strategies and transform domains to optimize system performance in demanding communication environments.

**Fig 14:**
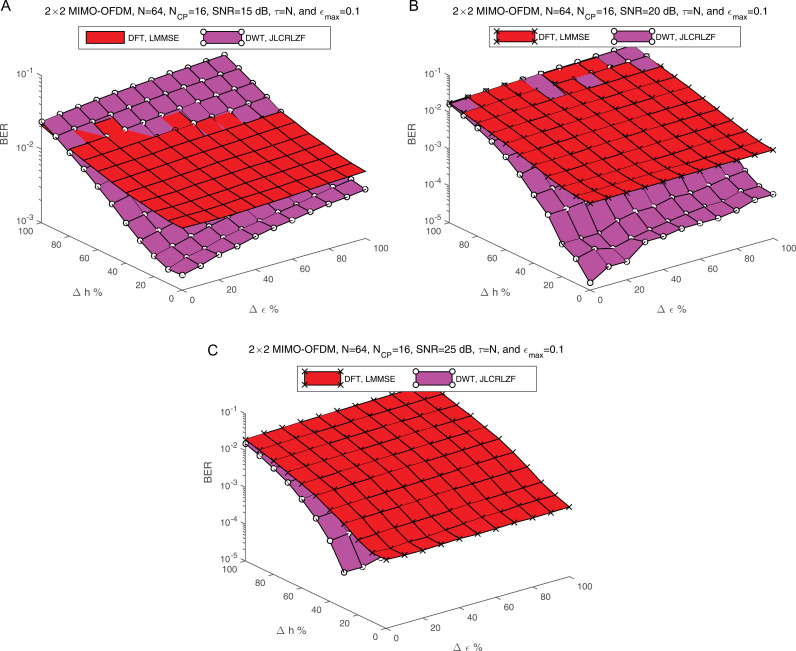
a: The BER against the estimation error percentage at SNR = 15 dB. b: The BER against the estimation error percentage at SNR = 20 dB. c: The BER against the estimation error percentage at SNR = 25 dB.

Fig 14 provides a detailed comparison of the BER performance for the proposed JLCRLZF-WDE and the LMMSE-FDE (based on DFT) under varying estimation error levels and SNR conditions. Each subfigure (i.e., Fig 14a, Fig 14b, and Fig 14c) illustrates the performance at SNR levels of 15 dB, 20 dB, and 25 dB, respectively. The analysis incorporates estimation errors ranging from 0% to 100%, which simulate inaccuracies in co-CFO and Rayleigh fading channel coefficients. These errors are critical in real-world scenarios, where imperfect channel estimation and synchronization often degrade system performance. By including these factors, the results offer a practical evaluation of the robustness of the equalizers. The proposed JLCRLZF-WDE consistently demonstrates superior performance across all scenarios, particularly at higher SNR levels and larger estimation errors (50%-100%). This resilience stems from its WDE, which provides better time-frequency localization and more effective interference mitigation than the frequency domain used by the LMMSE-FDE. The JLCRLZF-WDE shows remarkable stability at SNRs of 20 dB and 25 dB, maintaining low BER despite significant estimation errors, which highlights its adaptability for high-quality communication systems. In contrast, the LMMSE-FDE performs reasonably well at low estimation error levels (0%-20%) but suffers a steep decline as errors increase, particularly at lower SNRs like 15 dB, where noise and interference are more prominent. Overall, these results underscore the effectiveness of the proposed JLCRLZF-WDE compared to the conventional LMMSE-FDE. The JLCRLZF-WDE is a robust and versatile solution, capable of maintaining low BER across a wide range of SNRs and estimation error scenarios, even under extreme conditions. Its resilience to channel imperfections and frequency offset errors makes it a strong candidate for modern communication systems, where reliability and high performance are essential. Conversely, the LMMSE-FDE’s performance limitations, especially in high-error environments, highlight the need for more advanced equalization strategies like those employed in the JLCRLZF-WDE.

## VI. Complexity analysis

The complexity estimations of various equalizers are presented in this section. Half flops can be used to execute real multiplication, addition, and division in a single operation [36]. The quantity of floating-point operations completed in a second is measured in “flops”. The total count of operations and floating-point operations (flops) related to different mathematical operation situations is shown in Table 4. Meanwhile, Table 4 outlines the computational steps involved in basic mathematical operations, such as addition, multiplication, and division, for both real and complex numbers. It highlights how the complexity increases when dealing with complex arithmetic due to the need for additional operations, such as handling real and imaginary components. This table serves as a foundation for understanding the computational cost of individual operations used in more extensive processes. Table 5 shifts focus to full-matrix computations, covering operations like matrix-matrix multiplication, matrix inversion, and matrix-vector multiplication. It distinguishes between operations on real and complex matrices, emphasizing how the type of matrix and the operation performed influence the overall computational cost. This table is particularly useful for evaluating the efficiency of matrix-based algorithms in various applications. So, for each of the subsequent equalizations, compute the total number of operations and flops for a 2 × 2 MIMO-OFDM network. It should be noted that all equalizers are implemented for complete matrix implementations (i.e., τ=N).

**Table 4:** The number of flops related to different mathematical operations [36].

	Number of				
Process	×	±	÷	Operations	Flops
p±q	0	1	0	1	0.5
p×q	1	0	0	1	0.5
p÷q	0	0	1	1	0.5
$p+\left(q\pm jw\right)$	1	1	0	1	0.5
$p\times \left(q\pm jw\right)$	2	0	0	2	1
$\left(p\pm jq\right)\pm \left(w\pm jz\right)$	0	2	0	2	1
$\left(p\pm jq\right)\times \left(w\pm jz\right)$	4	2	0	6	3
$1÷\left(p\pm jq\right)$	0	0	1	2	1

**Table 5:** The number of flops related to different full-matrix operations [37].

Process	Description	Full-matrix computation						
		Number of complex		Number of real-complex			Total number of	
		×	∓	×	∓	÷	Operations	Flops
A∓B	A,B ∈ℂ^*N* × N^	---	$2N^{2}$	---	---	---	$2N^{2}$	$N^{2}$
A.B		$N^{3}$	$N^{3}$	---	---	---	$8N^{3}$	$4N^{3}$
$A^{-1}$		$N^{3}$	$N^{3}$	$N^{2}$	---	*N*	$8N^{3}+2N^{2}+N$	$4N^{3}+N^{2}+N/2$
A.U		$N^{2}$	$N^{2}$	---	---	---	$8N^{2}$	$4N^{2}$
A.V	$V\in {\mathbb{R}}^{N\times 1}$	---	---	$N^{2}$	$N^{2}$	---	$4N^{2}$	$2N^{2}$
V.M	*M* ∈ *ℝ* ^N × N^	---	---	$N^{2}$	$N^{2}$		$2N^{2}$	$N^{2}$

However, the complexity analysis aims to estimate the number of flops required, beginning with the generation of the polar-NRZ bits and continuing through to the estimation of the recovered bits. The overall processing is quite consistent, but differences arise based on the type of equalizer used (i.e., LZF, LMMSE, or JLCRLZF) and the domain of the equalizer (i.e., FDE or WDE).

The following analysis estimates the flop counts for various common processes at both the transmitter and receiver sides

### Transmitter side

At the transmitter, the generated polar-NRZ bits are multiplied by the IDWT or the IDFT matrix [38].

- For IDWT: The multiplication of a vector by a matrix (real-real) requires approximately flops.
- For IDFT: The multiplication of a vector by a matrix (real-complex) requires approximately 2 flops.

### Receiver side

At the receiver, the flops count depends on the equalization method used:

- For WDE: The multiplication of a vector by a matrix (complex-real) requires around 2 flops. Additionally, the operation and solution of the WDE contribute extra flops, but the exact count for solving the WDE depends on its specific implementation.
- For FDE with DWT: The receiver includes an extra IDFT and DFT operation. Each of these operations involves a vector-matrix multiplication (complex-complex), requiring approximately 4flops for each operation. Additionally, the operation and solution of the FDE contribute extra flops, depending on the specific FDE algorithm.
- For IDFT at the transmitter: The multiplication of a vector by a matrix (real-complex) requires 2 flops. On the receiver side, the DFT operation involves a vector-matrix multiplication (complex-complex), which requires approximately 4 flops, along with the operation and solution of the FDE.

### Total flops for each System

Now, let’s compute the total number of flops for each system, considering the respective equalizer and equalizer domain:

1. System with WDE and IDWT at transmitter:
  - Transmitter: IDWT (real-real vector-matrix multiplication): flops.
  - Receiver: WDE (complex-real vector-matrix multiplication): flops, plus the WDE operation and solution (which can vary depending on implementation, but let’s assume a constant factor ). Thus, the total flops: 3+.
2. System with FDE and IDWT at transmitter:
  - Transmitter: IDWT (real-real vector-matrix multiplication): flops.
  - Receiver: FDE with DWT, IDFT, and DFT operations. For each, the vector-matrix (complex-complex) multiplication requires 4 flops, and the vector-matrix (complex-real) multiplication requires 2 flops. Thus, for DWT and both IDFT, DFT, the total flops are $10N^{2}$ flops, plus the FDE operation and solution (with a constant factor $\gamma_{\text{FDE}}$). Thus, the total flops: $11N^{2}+\gamma_{\text{FDE}}$}
3. System with FDE and IDFT at Transmitter:
  - Transmitter: IDFT (real-complex vector-matrix multiplication): $2N^{2}$ flops.
  - Receiver: FDE with DFT operations: The vector-matrix (complex-complex) multiplication requires $4N^{2}$ flops, plus the FDE operation and solution ($\gamma_{\text{FDE}}$).

Total flops: $5N^{2}+\gamma_{\text{FDE}}$

In each case, the constants $\gamma_{\text{WDE}}$. and $\gamma_{\text{FDE}}$ represent the additional flops involved in solving the WDE and FDE algorithms. These constants (i.e., $\gamma_{\text{WDE}}$ or $\gamma_{\text{FDE}}$) depend on the specific algorithm and its implementation (i.e., the BMA bandwidth).

### A. The LZF

The LZF general solution matrix may be expressed as follows:


$$C_{\text{LZF}}={\left(\Pi^{H}\Pi \right)}^{-1}\Pi^{H}$$ (24)

where$\Pi \in {\mathbb{C}}^{iN\times jN}$contains the IRM in the case of WDE, or Fourier transform of the IRM in the case of FDE. According to Eq. (24), entails the production of two complex matrices via matrix multiplication and matrix inversion. For simplicity, we start with i=j=1. Multiplying two complex matrices requires $16N^{3}$ operations, according to Tables 4 and 5. Complex matrix inversion necessitates $8N^{3}+2N^{2}+N$ operations. The matrix inversion may be validated using the Singular Value Decomposition (SVD) concept [37]. The LZF solution matrix is multiplied by the received complex vector after the removal of the CP. This step involves a vector-matrix multiplication (complex-complex), which requires $8M^{2}$ operations. Consequently, the total number of operations to construct the LZF equalizer solution matrix and multiply it by the received vector amounts to $24N^{3}+10N^{2}+N$, corresponding to $12N^{3}+5N^{2}+0.5N$ flops. Note that, the primary general challenge of LZF equalization is the noise increase problem produced by direct matrix inversion.

### B. The LMMSE

The LMMSE’s solution matrix may be expressed as follows:


$$C_{\text{LMMSE}}={\left(\Pi^{H}\Pi +\frac{{\text{R}}_{\text{z}}}{\sigma_{x}^{2}}I_{jN\times jN}\right)}^{-1}\Pi^{H}$$ (25)

where the SNR value is represented by the term $\left(\frac{\sigma_{x}^{2}}{{\text{R}}_{z}}\right)$, the AWGN covariance matrix is represented by ${\text{R}}_{\text{z}}=E\left\{z.z^{H}\right\}$, the expectation of  #  is represented by $E\left\{\#\right\}$, and the transmitted signal power is represented by $\sigma_{\text{x}}^{2}$. The discrepancy between Eqs. (24, 25) represents an estimate of the SNR values, that might have caused a processing time delay in comparison to LZF besides the addition of the SNR to the identity matrix $I_{jN\times jN}$. As a result, the LMMSE needs $12N^{3}+5N^{2}+N$flops in addition to the SNR computation.

Now, let’s calculate the number of operations/flops for various equalizer types for a general i×jMIMO configuration as follows:

#### 1. *The LZF-FDE based on the DWT.*

As previously mentioned, at the transmitter side, each branch requires$N^{2}$ flops. Therefore, foritransmitted branches, the total number of flops is $iN^{2}$ flops. On the receiver side,jbranches are needed at the receiver side, which requiresjIDFTs,jDFTs, andjDWTs, in addition to the solution matrix for the LZF equalizer. The computational cost of the requiresjIDFTs,jDFTs, andjDWTs is$j10N^{2}$ flops. The solution matrix of the LZF equalizer requires$\gamma_{\text{FDE}}=12{\left(jN\right)}^{3}+5{\left(jN\right)}^{2}+0.5\left(jN\right)$ flops. Therefore, for an i×jMIMO configuration, the total number of flops is $12{\left(jN\right)}^{3}+5{\left(jN\right)}^{2}+j10N^{2}+iN^{2}+0.5\left(jN\right)$.

#### 2. *The LMMSE-FDE based on the DWT.*

The number of the flops for the case of LMMSE-FDE based on DWT is similar than that of the LZF-FDE based on DWT in addition to the SNR value calculation and jN flops. Thus, this corresponds to $12{\left(jN\right)}^{3}+5{\left(jN\right)}^{2}+j10N^{2}+iN^{2}+1.5\left(jN\right)$ flops.

#### 3. *The LZF-WDE.*

The LZF-WDE requires the same number of flops as the LZF-FDE, but without the need for DFT and IDFT processing. This is because the LZF-WDE uses the DWT instead. Therefore, the total computational cost is given by $12{\left(jN\right)}^{3}+5{\left(jN\right)}^{2}+j2N^{2}+iN^{2}+0.5\left(jN\right)$ flops.

#### 4. *The LMMSE-WDE.*

Similarly, the LMMSE-WDE requires the same number of flops as the LMMSE-FDE, but without the need for DFT and IDFT processing, as it uses the DWT instead. Therefore, the computational cost is $12{\left(jN\right)}^{3}+5{\left(jN\right)}^{2}+j2N^{2}+iN^{2}+1.5\left(jN\right)$ flops, in addition to the processing time required for SNR estimation.

#### 5. *The JLCRLZF-WDE.*

Similarly, the JLCRLZF-WDE needs the same number of flops as that of the LMMSE-WDE, which $12{\left(jN\right)}^{3}+5{\left(jN\right)}^{2}+j2N^{2}+iN^{2}+1.5\left(jN\right)$ flops with no SNR calculation required.

#### 6. *The LZF-FDE based on the DFT.*

At the transmitter side, each branch requires $2N^{2}$ flops. Therefore, for itransmitted branches, the total number of flops is $i2N^{2}$ flops. On the receiver side, jbranches are needed at the receiver side, which requires j DFTs, in addition to the solution matrix for the LZF equalizer. The computational cost of the requires j DFTs is $j4N^{2}$ flops. The solution matrix of the LZF equalizer requires $\gamma_{\text{FDE}}=12{\left(jN\right)}^{3}+5{\left(jN\right)}^{2}+0.5\left(jN\right)$ flops. Therefore, for an i×jMIMO configuration, the total number of flops is $12{\left(jN\right)}^{3}+5{\left(jN\right)}^{2}+j4N^{2}+i2N^{2}+0.5\left(jN\right)$.

#### 7. *The LMMSE-FDE based on the DFT.*

Likewise, $12{\left(jN\right)}^{3}+5{\left(jN\right)}^{2}+j4N^{2}+i2N^{2}+1.5\left(jN\right)$ flops are needed for the LMMSE-FDE based on DFT in addition to the SNR value calculation.

Table 6 presents the computational requirements, expressed as the number of flops, for various equalization methods in an i×j MIMO system configuration. The table compares different combinations of equalizers (e.g., LZF, LMMSE, JLCRLZF), transform methods (e.g., DWT or DFT), and equalization domains (e.g., WDE or FDE). It provides a detailed breakdown of the complexity associated with each approach, highlighting how the choice of equalizer, transform, and domain affects the overall computational demand. This comparison is essential for evaluating the trade-offs between computational efficiency and system performance in different MIMO equalization schemes.

**Table 6:** The number of flops for different equalization methods.

Type of			Number of flops
Equalizer	Transform	Equalization	
LZF	DWT	WDE	$12{\left(jN\right)}^{3}+5{\left(jN\right)}^{2}+j2N^{2}+iN^{2}+0.5\left(jN\right)$
LMMSE	DWT	WDE	$12{\left(jN\right)}^{3}+5{\left(jN\right)}^{2}+j2N^{2}+iN^{2}+1.5\left(jN\right)$
LZF	DWT	FDE	$12{\left(jN\right)}^{3}+5{\left(jN\right)}^{2}+j10N^{2}+iN^{2}+0.5\left(jN\right)$
LMMSE	DWT	FDE	$12{\left(jN\right)}^{3}+5{\left(jN\right)}^{2}+j10N^{2}+iN^{2}+1.5\left(jN\right)$
LZF	DFT	FDE	$12{\left(jN\right)}^{3}+5{\left(jN\right)}^{2}+j4N^{2}+i2N^{2}+0.5\left(jN\right)$
LMMSE	DFT	FDE	$12{\left(jN\right)}^{3}+5{\left(jN\right)}^{2}+j4N^{2}+i2N^{2}+1.5\left(jN\right)$
JLCRLZF	DWT	WDE	$12{\left(jN\right)}^{3}+5{\left(jN\right)}^{2}+j2N^{2}+iN^{2}+1.5\left(jN\right)$

**Fig 15:**
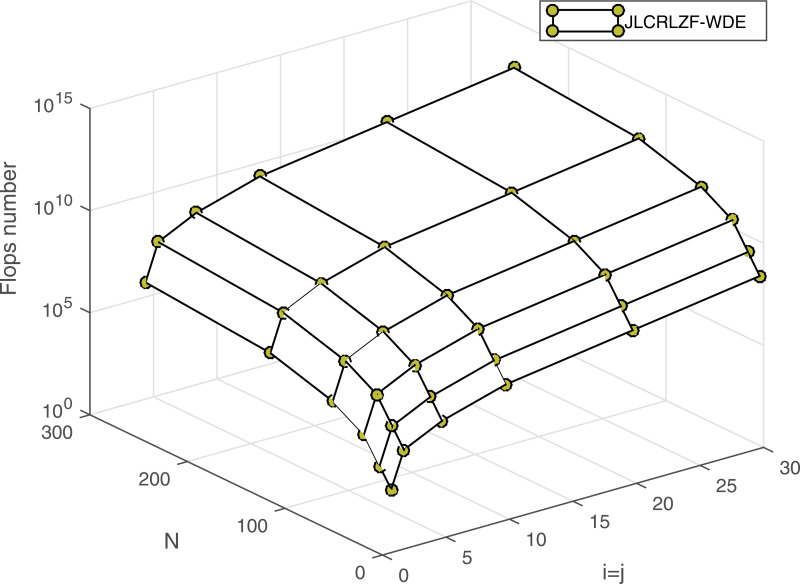
The flops number at different values of the channel configuration and length of the transmitted bits.

As shown in Table 6, all the systems presented exhibit a computational complexity of order $N^{3}$, which implies that their computational demands become comparable for large values of *N*. To better understand the impact of MIMO configuration and data length *N* on the computational load, we analyze the proposed JLCRLZF-WDE system. Fig 15 illustrates the number of flops for various i×j MIMO configurations and different lengths of the transmitted data vector *N*, assuming i=j for simplicity. The figure highlights a clear trend: the number of flops increases as *N*, *i*, or *j* grows. This increase is expected, as larger data lengths and more complex MIMO configurations demand greater computational resources for processing. The analysis underscores the importance of balancing computational efficiency and performance when scaling MIMO systems, particularly for scenarios involving high data rates or large-scale antenna arrays.

The percentage reduction in simulated time for each type of equalizer compared to the proposed JLCRLZF-WDE is as follows:


$$\text{η\%}=\frac{t_{2}-t_{1}}{t_{1}}\%$$ (26)

where $t_{1}$represents the simulation time of the proposed JLCRLZF-WDE and $t_{2}$ represents the simulation time of the comparable equalizer. The negative value of the simulation time in Table 7 indicates that the comparative equalizer takes less time than the reference, which is the proposed JLCRLZF-WDE. Table 7 compares various equalization methods for a 2 × 2 MIMO configuration, focusing on simulation time, efficiency $\left(\text{η\%}\right)$, and the inclusion of SNR estimation. The methods are categorized by the type of equalizer (e.g., LZF, LMMSE, JLCRLZF), the transform method (DWT or DFT), and the equalization domain (WDE or FDE). LZF with DWT and WDE demonstrates one of the shortest simulation times (21.58 ms), while LMMSE with DWT and FDE has the longest (24.83 ms), indicating that the choice of equalizer and domain significantly impacts processing speed. Regarding efficiency, LZF with DWT and FDE shows the lowest (-4.98%), whereas LMMSE with DWT and FDE achieves the highest (12.4%), showcasing the trade-offs between computational cost and performance. Additionally, some methods, such as LMMSE with DWT and WDE or JLCRLZF with DWT and WDE, incorporate SNR estimation, offering better adaptability to changing channel conditions. Overall, for a 2 × 2 MIMO configuration, the selection of an equalization method depends on balancing computational demands, efficiency, and the need for SNR estimation.

**Table 7:** The average simulated time for different equalization methods.

Type of			Simulation time for each run (m sec)	η%	SNR Est.
Equalizer	Transform	Equalization			
LZF	DWT	WDE	21.58	-2.31	×
LMMSE	DWT	WDE	22.79	3.17	✓
LZF	DWT	FDE	20.99	-4.98	×
LMMSE	DWT	FDE	24.83	12.4	✓
LZF	DFT	FDE	21.91	-0.81	×
LMMSE	DFT	FDE	22.37	1.27	✓
JLCRLZF	DWT	WDE	22.09	0.00	×

After discussing the complexity analysis, we believe that certain modifications related to the concept of BMA could be implemented to reduce the computational complexity of the proposed JLCRLZF-WDE in some way as many zeros are present in the matrix to be used for equalization process. Incorporating BMA techniques may lead to more efficient computations, optimizing the overall performance. Additionally, there are several other limitations in the current approach that could be addressed and optimized in future work as Adaptive techniques. The proposed JLCRLZF-WDE could benefit from adaptive algorithms that adjust to the specific conditions of the channel or signal environment. For example, an adaptive approach could dynamically adjust the degree of approximation in the BMA or the size of the matrix, depending on the SNR or the channel conditions, leading to a more efficient computational approach in varying environments.

The practical relevance of the proposed JLCRLZF-WDE can be seen in its potential applications in real-world wireless communication systems. For instance, in millimeter-wave communication scenarios, such as 5G and beyond, the JLCRLZF-WDE’s ability to mitigate co-CFO and ISI while reducing computational complexity makes it highly suitable for high-frequency, high-speed networks with dense deployments. Similarly, in WSNs, where energy efficiency and reliable communication are critical, the proposed approach enhances spectral efficiency and reduces power consumption, aligning with the operational constraints of such networks. Furthermore, its simplicity and computational efficiency make it feasible for hardware implementation on devices like Field-Programmable Gate Arrays (FPGAs) and Digital Signal Processors (DSPs). By addressing challenges like imperfect channel estimation and high Doppler effects, the JLCRLZF-WDE demonstrates robust performance under practical conditions, as highlighted in updated simulations. These capabilities make it a strong candidate for applications in vehicular communication systems, scalable massive MIMO systems, and other next-generation wireless technologies, where low latency and resilience to interference are essential.

## VII. Conclusion

In this study, we introduced the JLCRLZF-WDE for MIMO-DWT-OFDM systems and compared its performance against various equalizers through multiple evaluations. The proposed JLCRLZF-WDE effectively integrates the equalization and co-CFO compensation processes. It is specifically designed to address challenges such as co-CFO, ISI, co-channel interference, and noise. To achieve a BER of 10^-4^, the traditional LMMSE-FDE based on DWT necessitates an additional SNR of approximately 12.03 dB. Notably, the JLCRLZF-WDE demonstrates a significant reduction in simulation time, approximately 12.4% less than the conventional LMMSE-FDE based on DWT. Furthermore, computer simulations indicate that the JLCRLZF-WDE offers improved computational efficiency compared to other equalizers. This balance between complexity and BER performance presents a viable option for future advancements in MIMO-DWT-OFDM systems.

For future work, the proposed JLCRLZF-WDE can be extended and optimized for other types of fading channels, such as Rician or Nakagami, to further validate its performance in diverse communication environments. Future work on the JLCRLZF-WDE method could explore several optimizations to enhance its performance. These include integrating advanced channel estimation algorithms to improve equalization accuracy, especially in challenging wireless environments with severe impairments. Additionally, refining the BMA by exploiting matrix sparsity could reduce computational complexity while maintaining accuracy. The inclusion of adaptive algorithms would allow the method to dynamically adjust to varying channel conditions, improving efficiency across diverse environments. Future research could also evaluate alternative wavelet transforms, such as biorthogonal or symlet wavelets, for better time-frequency localization. Expanding the method to support Non-Orthogonal Multiple Access (NOMA) would enable its application in next-generation communication systems. Investigating non-linear equalization techniques, such as decision-feedback equalization or neural network-based approaches, could further enhance performance in highly non-linear channels. Finally, extending the framework to accommodate higher-order MIMO systems and considering its performance under 5G or beyond networks would offer insights into its scalability and relevance to next-generation systems.

## References

[1] Hassan ES. Multi-carrier communication systems with examples in MATLAB®: A new perspective. vol. 47, CRC Press, Taylor & Francis. 2019, p. 26–35, ISBN 9781498735322.

[2] Trivedi VK, Ramadan K, Kumar P, Dessouky MI, Abd El-Samie FE. Enhanced OFDM-NOMA for next generation wireless communication: A study of PAPR reduction and sensitivity to CFO and estimation errors. AEU - Int J Electron Commun. 2019;102:9–24. doi: 10.1016/j.aeue.2019.01.009

[3] Hassan ES, Alharbi AA, Oshaba AS, El-Emary A. enhancing smart irrigation efficiency: a new WSN-based localization method for water conservation. Water. 2024;16(5):672. doi: 10.3390/w16050672

[4] Tang Z. OFDM communication system based on FPGA. In 2023 3rd Asia-Pacific Conference on Communications Technology and Computer Science (ACCTCS). 2023, p. 666–70.

[5] Sharma V, Sergeyev S, Kaur J. Transmission, adaptive 2×2 MIMO employed wavelet-ofdm-radio over fibre. IEEE Access. 2020;8 23336–45.

[6] Li A, Shieh W, Tucker RS. Wavelet packet transform-based OFDM for optical communications. J Lightwave Technol. 2010;28(24):3519–28.

[7] Wils M, Sharma M, Moonen M. Per-wavelet equalization for discrete wavelet transform based multi-carrier modulation systems. IEEE Open J Signal Process. 2023;4 52–60. doi: 10.1109/ojsp.2023.3242122

[8] Ramadan K, Dessouky MI, Abd El-Samie FE. Non-linear equalisation and CFO compensation for MIMO-OFDM communication systems based on DWT. Int J Electron. 2020;108(1):115–38. doi: 10.1080/00207217.2020.1756458

[9] Vishvaksenan KS, Natarajan K, Sathiyarajan M, Vengatraman M. Performance of MIMO MC-CDMA system for DWT technique based colour image transmission over correlated frequency-selective channel. In 2014 International Conference on Communication and Signal Processing, Melmaruvathur, India, 2014.

[10] Parveen N, Abdullah K, Islam R, Islam Boby R. Performance of MIMO DWT for Millimeter Wave Communication System. In 2019 IEEE 14th Malaysia International Conference on Communication (MICC), Selangor, Malaysia. 2019.

[11] Simon J, Prabakaran N. Telemedicine application based on MIMO-OFDM system along with DWT. In Proceedings of the 2020 Fourth International Conference on I-SMAC (IoT in Social, Mobile, Analytics and Cloud), Palladam, India. 2020.

[12] Wachid AR, Asfani DA, Negara IMY, Ksatria AB. Design and implementation of arcing detection on low voltage with evaluation of discrete wavelet transform. In 2024 International Seminar on Intelligent Technology and Its Applications (ISITIA), Mataram, Indonesia. 2024, p. 536–41.

[13] Rana ASTK. Comparisons of wavelets and algorithms based on wavelets and comparing the results with JPEG. In Proceedings of the 2017 International Conference on Energy, Communication, Data Analytics and Soft Computing (ICECDS), Chennai, India. 2017.

[14] Hassan ES, Dessouky AM, Fathi H, Salama GM, Oshaba AS, El-Emary A, et al. Improved hybrid approach for enhancing protein-coding regions identification in DNA sequences. Current Bioinformatics. 2025;20(3):208–28. doi: 10.2174/0115748936287244240117065325

[15] Singhal R p. s. a. M t. A. Comparison of different wavelets for watermarking of colored images. In 2011 3rd International Conference on Electronics Computer Technology, Kanyakumari, India. 2011.

[16] Todorova RPM. Influence of the sampling period in the identification of a dual-mass DC electromechanical system using Symlet wavelets. In 2020 International Conference Automatics and Informatics (ICAI), Varna, Bulgaria. 2020.

[17] Sridhar DNS. P. r. k. K. V. R. Coiflets, artificial neural networks and predictive coding based hybrid image compression methodology. In 2014 2nd International Conference on Devices, Circuits and Systems (ICDCS). 2014.

[18] He H, Wan M, Xu Y, Kong X, Liu Z, Chen Q, et al. WTAPNet: Wavelet Transform-Based Augmented Perception Network for Infrared Small-Target Detection. IEEE Trans Instrum Meas. 2024;73:1–17. doi: 10.1109/tim.2024.3476549

[19] TODOROVA RPM. Biorthogonal wavelet filtration of signals used in the industrial automation systems. In 2019 16th Conference on Electrical Machines, Drives and Power Systems (ELMA), Varna, Bulgaria. 2019.

[20] Leontiev AN. The use of discrete Meyer wavelet for speech segmentation. In 2019 International Multi-Conference on Industrial Engineering and Modern Technologies (FarEastCon), Vladivostok, Russia. 2019.

[21] Li X, Zhang P, Hui E, Chen G. Based on Embedded Zerotree Wavelet Coding Data Compression Algorithm Research of NC Machine Tool Control System. In 2019 4th International Conference on Mechanical, Control and Computer Engineering (ICMCCE), Hohhot, China. 2019, p. 936–9364. doi: 10.1109/icmcce48743.2019.00211

[22] Cho AS a. WAPY. Coding the wavelet spatial orientation tree with low computational complexity. In Data Compression Conference, Snowbird, UT. 2005.

[23] Lamsrichan TSP. Embedded color image coding with context adaptive wavelet difference reduction. 2009 International Symposium on Intelligent Signal Processing and Communication Systems (ISPACS), Kanazawa, Japan. 2009.

[24] Usman A, Baig S, Umer T, Ding Z. Performance analysis of discrete wavelet transform for downlink non-orthogonal multiple access in 5G networks. IET Communications. 2020;14(10):.

[25] Dang S, Zhou J, Shihada B, Alouini M. Toward spectral and energy efficient 5G networks using relayed OFDM with index modulation. Frontiers in Communications and Networks. 2021;2:635295. doi: 10.3389/frcmn.2021.635295

[26] Mori C, Sawahasi M, Miki N, Suyama S. Performance of iterative decision feedback channel estimation in turbo FDE for DFT-spread OFDM. IEICE Trans Commun. 2024:1–13. doi: 10.23919/transcom.2024ebp3052

[27] Yılmaz G, Yılmaz AÖ. Quasi-Newton FDE in one-bit pseudo-randomly quantized massive MIMO-OFDM systems. IEEE Commun Lett. 2024;28(4):917–21. doi: 10.1109/lcomm.2024.3363878

[28] Ou J, Zhou B, Zhang X, Liao X. Performance improvement of multi-level redundant discrete wavelet transform OFDM system by using LDPC encoding and belief propagation algorithm. In 2023 8th International Conference on Computer and Communication Systems (ICCCS), Guangzhou - China. 2023, p. 269–73. doi: 10.1109/icccs57501.2023.10151120

[29] Chen W, Sai F, Huang Z. Comparative Analysis of Performance of DWT-OFDM and DFT-OFDM. In 2023 3rd Asia-Pacific Conference on Communications Technology and Computer Science (ACCTCS), Shenyang, China. 2023 684–90. doi: 10.1109/acctcs58815.2023.00129

[30] Zheng T, He C, Jing L, Dong X, Cao X, Yan Q. Adaptive Equalization with Interference Reconstruction and Cancellation Based on MSER Criterion for OTFS System. In 2024 IEEE International Conference on Signal Processing, Communications and Computing (ICSPCC), Bali, Indonesia. 2024 1–5. doi: 10.1109/icspcc62635.2024.10770334

[31] Chafii M, Harbi YJ, Burr AG. Wavelet-OFDM vs. OFDM: Performance comparison. In 2016 23rd International Conference on Telecommunications (ICT), Thessaloniki, Greece. 2016.

[32] Cao Z, Tureli U, Yao Y-D. Low-Complexity Orthogonal Spectral Signal Construction for Generalized OFDMA Uplink With Frequency Synchronization Errors. IEEE Trans Veh Technol. 2007;56(3):1143–54. doi: 10.1109/tvt.2007.895601

[33] Swamy VN, Rigge P, Ranade G, Nikolic B, Sahai A. Wireless Channel Dynamics and Robustness for Ultra-Reliable Low-Latency Communications. IEEE J Select Areas Commun. 2019;37(4):705–20. doi: 10.1109/jsac.2019.2900784

[34] Dent P, Bottomley GE, Croft T. Jakes fading model revisited. Electron Lett (UK). 1993;29(13):1162–3. doi: 10.1049/el:19930777

[35] 3rd Generation Partnership Project. 3rd Generation Partnership Project, 3GPP TS 25.101 – Technical Specification Group Radio Access Network; User Equipment Radio Transmission and Reception (Release 7), Section B.2.2. Sep 2007.

[36] Elshokry AM. Complexity and performance evaluation of detection schemes for spatial multiplexing MIMO systems. Islamic University Gaza, Palestine, 2010.

[37] Zhen Z, Zhang Y, Liu X, Ling Y, Yang M. Numerical computation, symbolic computation and result analysis of Jordan decomposition of time-varying matrices. In 2020 IEEE International Conference on Mechatronics and Automation (ICMA). 2020, p. 1529–35. doi: 10.1109/icma49215.2020.9233728

[38] Li B, Jiang Z, Chen J. On performance of sparse fast fourier transform algorithms using the flat window filter. IEEE Access. 2020;8:79134–46. doi: 10.1109/access.2020.2989327

