# Correction: Enhanced DWT-OFDM communication system using wavelet domain equalizer with Co-CFO

**DOI:** 10.1371/journal.pone.0333324

**Published:** 2025-09-29

**Authors:** Khaled Ramadan, Emad S. Hassan

The authors issue this correction to clarify that the AI-based MATLAB Signal Processing Toolbox was used for data transformation and computation during the study.

The Simulation results and analysis section is updated to include the following statement: The MATLAB Signal Processing Toolbox AI-based tool was used for signal decomposition and equalizer coefficient computation in this study.

The authors apologize for the error in the published article.
